# Dietary Restriction Mitigates Vascular Aging, Modulates the cGAS‐STING Pathway and Reverses Macrophage‐Like VSMC Phenotypes in Progeroid DNA‐Repair‐Deficient Ercc1^Δ^ ^/−^ Mice

**DOI:** 10.1111/acel.70062

**Published:** 2025-04-25

**Authors:** S. J. M. Stefens, J. van der Linden, J. M. Heredia‐Genestar, R. M. C. Brandt, S. Barnhoorn, I. Nieuwenhuizen‐Bakker, N. van Vliet, J. H. M. Odijk, Y. Ridwan, D. Stuijts, M. Batenburg, J. H. J. Hoeijmakers, R. Kanaar, J. Essers, I. van der Pluijm

**Affiliations:** ^1^ Department of Molecular Genetics Erasmus University Medical Center Rotterdam the Netherlands; ^2^ AMIE Core Facility Erasmus University Medical Center Rotterdam the Netherlands; ^3^ Princess Máxima Center for Pediatric Oncology Utrecht the Netherlands; ^4^ Oncode Institute Utrecht the Netherlands; ^5^ Institute for Genome Stability in Aging and Disease, Cologne Excellence Cluster for Cellular Stress Responses in Aging‐Associated Diseases (CECAD) University of Cologne Cologne Germany; ^6^ Department of Radiotherapy Erasmus University Medical Center Rotterdam the Netherlands; ^7^ Department of Vascular Surgery, Cardiovascular Institute Erasmus University Medical Center Rotterdam the Netherlands

**Keywords:** cGAS‐STING, dietary restriction, DNA damage, Ercc1, intervention, macrophage‐like VSMCs, vascular aging

## Abstract

Aging is a major risk factor for cardiovascular diseases, and the accumulation of DNA damage significantly contributes to the aging process. This study aimed to identify the underlying molecular mechanisms of vascular aging in DNA‐repair‐deficient progeroid *Ercc1* ^*Δ/−*^ mice and to explore the therapeutic effect of dietary restriction (DR). RNA sequencing analysis revealed that DR reversed gene expression of vascular aging processes, including extracellular matrix remodeling, in the *Ercc1* ^*Δ/−*^ aorta. Notably, this analysis indicated the presence of macrophage‐like vascular smooth muscle cells (VSMCs) and suggested cGAS‐STING pathway activation. The presence of macrophage‐like VSMCs and increased STING1 expression were confirmed in *Ercc1* ^*Δ/−*^ aortic tissue and were both reduced by DR. In vitro, cisplatin‐induced DNA damage activated the cGAS‐STING pathway in *Ercc1* ^*Δ/−*^ VSMCs but not in wildtype VSMCs. These findings identify the involvement of the cGAS‐STING pathway in DNA damage‐driven vascular aging and underscore the therapeutic benefits of DR for vascular aging. Furthermore, upstream regulator analysis revealed compounds that may replicate the beneficial effects of DR, providing promising leads for further investigation.

## Graphical abstract

Dietary restriction mitigates vascular aging in DNA‐repair‐deficient *Ercc1* ^*Δ/−*^ mice by reducing extracellular matrix remodeling, inflammation, oxidative stress, and switching of vascular smooth muscle cells to a macrophage‐like phenotype. Our findings suggest that the cGAS‐STING pathway plays a key role in vascular aging and that the benefits of dietary restriction may result from reducing its activation. Created in BioRender. Van der pluijm, I. (2025) https://BioRender.com/iwpqncu.

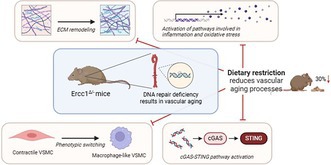

## 1 Introduction

Aging is a major risk factor for cardiovascular diseases (CVDs), including hypertension, atherosclerosis, myocardial infarction, and stroke. The proportion of elderly in the total population continues to rise, with projections suggesting that by 2050 around 16% of the global population will be 65 years or older (Zhao et al. 2024). This demographic shift is a significant factor behind the expected increase in global CVD mortality of 73.4% between 2025 and 2050 (Zhao et al. 2024). This underscores the importance of better understanding the underlying mechanisms of cardiovascular aging. One key factor contributing to aging is the accumulation of DNA damage (Schumacher et al. 2021). This is evident in rare human syndromes where deficiencies in proteins involved in specific DNA repair pathways result in premature multimorbidity and many features of accelerated aging (Jaspers et al. 2007; Rapin et al. 2000). Examples are Cockayne syndrome and Xeroderma Pigmentosum, in which patients lack the ERCC1‐XPF DNA repair endonuclease complex (Faridounnia et al. 2018). The ERCC1‐XPF complex is involved in multiple DNA repair pathways, including nucleotide excision repair, double strand break repair, and interstrand cross‐link repair, explaining why its deficiency causes a wide spectrum of aging features (Faridounnia et al. 2018). Consequently, the *Ercc1* ^*Δ/−*^ mouse model, which carries one loss‐of‐function allele and one hypomorphic allele encoding a truncated ERCC1 protein, exhibits multiple aging symptoms, including frailty, hearing loss, cognitive decline, osteoporosis, and overall reduced organ function, with a shortened lifespan of 4 to 6 months (Dollé et al. 2011; Vermeij et al. 2016; Weeda et al. 1997). Previous research has shown that the aorta of *Ercc1* ^*Δ/−*^ mice undergoes functional age‐related changes, including vasomotor dysfunction, increased vascular stiffness, and elevated blood pressure (Durik et al. 2012). Furthermore, we recently demonstrated that the aorta of these mice exhibits structural age‐related alterations, such as medial thickening, loss of cells due to apoptosis, phenotypic switching of vascular smooth muscle cells (VSMCs), and increased remodeling of the extracellular matrix (ECM) (van der Linden et al. 2024). These findings suggest that the accumulation of DNA damage plays a substantial role in vascular aging and possibly also in the development of CVDs. However, the precise molecular mechanisms underlying vascular aging in *Ercc1* ^*Δ/−*^ mice remain to be elucidated.

Given that CVDs are the leading cause of death globally, there is a critical need for improved therapeutic interventions to delay or possibly prevent cardiovascular aging. Dietary restriction (DR) is one such intervention that was shown to prolong lifespan and retard the onset of many age‐related symptoms in various species (Fontana et al. 2010). DR has also demonstrated strong beneficial effects in *Ercc1* ^*Δ/−*^ mice, delaying many aging characteristics and significantly extending their lifespan (Durik et al. 2012). Among others, DR rescued the age‐dependent decline in vasodilation in *Ercc1* ^*Δ/−*^ aorta segments. However, the underlying mechanisms of the therapeutic effect of DR on vascular aging in *Ercc1* ^*Δ/−*^ mice and its impact on the structural age‐related changes of the aorta have not been investigated yet. Furthermore, since DR is not easily translatable into clinical practice, understanding its mechanism could help identify pharmacological therapies that mimic this effect.

In this study, we aimed to identify the molecular basis of vascular aging in *Ercc1* ^*Δ/−*^ mice as well as the factors that drive the therapeutic benefit of DR in the aorta. First, we assessed the effect of DR on heart and aorta diameter and function through ultrasound imaging of *Ercc1* ^*Δ/−*^ mice and wildtype littermates subjected to either an ad libitum (AL) diet or a 30% DR regime. Next, we performed bulk mRNA sequencing on the aortas of these mice. Pathway analysis was performed to biologically interpret the data, and the findings were validated in aortic tissue and VSMCs in vitro.

## 2 Methods

### 2.1 Experimental Animals

*Ercc1* ^*Δ/−*^ mice were generated by crossing *Ercc1* ^*Δ/+*^ (in a pure C57BL6J or FVB background) with *Ercc1* ^*+/−*^ mice (in a pure FVB or C57BL6J background respectively), resulting in *Ercc1* ^*Δ/−*^ offspring with a genetically uniform F1 C57BL6J/FVB hybrid background. Wildtype (WT) F1 littermates served as controls. Animals were housed at the Animal Resource Center (Erasmus University Medical Centre), and all experiments were conducted in accordance with the Principles of Laboratory Animal Care and the guidelines approved by the Dutch Ethical Committee in full compliance with European legislation.

### 2.2 Dietary Restriction Experiment

All animals were bred and maintained on AIN93G synthetic pellets (Research Diet Services B.V.; gross energy content 4.9 kcal/g dry mass, digestible energy 3.97 kcal/g). Dietary restriction (DR) was initiated at 7 weeks of age by 10% food reduction (g/day). DR was increased weekly by 10% (g/day), until reaching 30% DR from 9 weeks of age onward. Mice in the *ad libitum* (AL) group were allowed unlimited access to AIN93G pellets. Mice were kept on an adjusted light–dark schedule (light from 12 pm to 12 am and dark from 12 am to 12 pm) to facilitate feeding just before the start of the dark (active) period, in order to avoid disruptions in the biological clock. Mice were individually housed to control food intake. At 6 weeks and 18–22 weeks of age, mice were sacrificed by CO_2_ inhalation for the described experiments.

### 2.3 Echocardiographic Measurements

To assess the effect of DR on aortic and cardiac function and geometry, echocardiographic measurements were conducted on 6‐week‐old and 20–22‐week‐old *Ercc1* ^*Δ/−*^ and *Ercc1* ^*+/+*^ mice that had either been on DR or an AL diet (*n* = 8–10 per group). Mice were anesthetized with 2.5% isoflurane and ventilated with 35% O_2_. Echocardiography of the ascending aorta and left ventricle (LV) was performed using a Vevo3100 (VisualSonics Inc., Toronto, Canada). Aortic and LV diameters, ejection fraction, stroke volume, and fractional shortening were obtained from M‐mode images. Aortic strain was calculated using the following formula: (aorta diameter in systole—aorta diameter in diastole)/aorta diameter in systole * 100%.

### 2.4 Molecular Imaging for MMP Activation

Mice were intravenously injected with matrix metalloprotease (MMP) Sense680 (Perkin Elmer, Waltham, MA, USA) at a concentration of 2 nmol per 25 g of body weight. After 24 h, aortas were collected and imaged using the Odyssey CLx imaging system (LI‐COR Biosciences, Lincoln, NE, USA). Probe intensity was quantified with Image Studio Lite version 5.20 (LI‐COR Biosciences, Lincoln, NE, USA). Probe intensity was normalized by dividing the intensity by the shape area, resulting in intensity/mm^2^ per aorta. *Ercc1* ^*Δ/−*^ aortas were normalized to the average of the WT littermate aortas that were scanned simultaneously. Measurements were performed on aortas of *Ercc1* ^*Δ/−*^ and *Ercc1* ^*+/+*^ mice at 18–20 weeks of age subjected to either DR or an AL diet (*n* = 5–7 per group).

### 2.5 RNA Sequencing

*Ercc1* ^*Δ/−*^ mice and their WT littermates were sacrificed at ages of 6 weeks or 22 weeks (*n* = 4–5 per group). Table S1 indicates the number of samples per group, including the sex of the mice used. Mice were perfused through the left ventricle with phosphate buffered saline (PBS) and the aortic arch was isolated. Aortic tissue was homogenized using the TissueLyser 24 (Shanghai Jing Xin). Total RNA and microRNA were isolated from the aortic arch using the miRNeasy Mini Kit (Qiagen). Library preparation and mRNA sequencing were performed at GenomeScan B.V. (Genomescan B.V., Leiden, The Netherlands). Libraries were sequenced on an Illumina NovaSeq 6000 platform, generating paired‐end reads (150 bp read length) with a sequencing depth of 20 million reads, including Unique Molecular Identifiers (UMIs).

### 2.6 Alignment and Quality Control

We used UMI‐Tools v1.1.2 to move the UMI barcodes from separate files to the read pair headers (Smith et al. 2017). The raw fastq files were pre‐processed with fastp v0.23.2 (Chen et al. 2018). Samples were then aligned using STAR v2.7.10a against the mouse reference genome mm39 with ENSEMBL v107 annotation (Dobin et al. 2012). After alignment, we applied UMI‐Tools to deduplicate reads originating from the same molecule and minimize gene‐length biases from PCR amplification. Quality Control (QC) was assessed using fastqc v0.11.9 and RSeQC v5.0.1 (Andrews 2010; Wang et al. 2012; Wang et al. 2016). MultiQC v1.15.dev0 was used to summarize the QC of these two programs and the different steps of the data processing (Ewels et al. 2016). Read counts overlapping the entire gene body were generated using htseq‐count v2.0.2 (Putri et al. 2022).

### 2.7 Differential Gene Expression Analysis

The resulting gene‐count matrices were analyzed using R v4.1.2 (Team RC 2021). One sample (003, WT, female, 22 weeks, AL) showed too low read counts and diverging expression profiles, and it was therefore excluded from subsequent analyses.

Differential gene expression between groups and conditions was analyzed using DESeq2 v1.34.0, with an adjusted *p*‐value threshold of 0.05 and a |log2FC| ≥ log2(1.2) (~ ± 0.26) (Love et al. 2014). Analyses were restricted to genes with > 10 read counts in more than 10 samples. The design model used was “design = ~group + sex”, where group represents the combination of age, genotype and diet, allowing the decoupling of the effect of sex from the comparisons of age, genotype and diet. Comparisons between samples at 6 weeks and 22 weeks of age were performed only for AL samples, excluding DR samples to avoid biases in gene expression levels. Conversely, analyses on the effect of DR used only 22‐week‐old samples.

NOTE: Part of this data has been used in a previously published study by our lab (van der Linden et al. 2024). This concerns only the data from AL mice, and the dataset itself has not been previously published.

### 2.8 Gene Set Enrichment Analysis

Gene Set Enrichment Analysis (GSEA) was performed using the R package fGSEA v1.20.0, and MSigDB (v2022.1) mouse Canonical Pathways were used as a reference (Subramanian et al. 2005; Castanza et al. 2023; Nishimura 2001; Milacic et al. 2023; Agrawal et al. 2023; Korotkevich et al. 2021). Each comparison between groups and conditions was analyzed separately using the log2 Fold Change of all detected genes. Genes not present in the data were excluded from the background.

### 2.9 Ingenuity Pathway Analysis

For biological interpretation of differential expression data, normalized expression values were uploaded into Ingenuity Pathway Analysis (IPA software version 94302991, QIAGEN, Redwood City, California, USA). An IPA core analysis was performed with significantly differentially expressed genes. Canonical pathways and upstream regulators were investigated, with upstream regulators selected based on *z*‐score cut‐offs (−2.0 ≤ *z*‐score ≥ 2.0) and *p*‐value ≤ 0.05. A *z*‐score ≥ 2 indicates predicted activation of the upstream regulator, and a *z*‐score ≤ −2.0 indicates predicted inhibition of the upstream regulator. Top upstream regulators were ranked based on their z‐scores.

### 2.10 Immunohistochemical Staining of Mouse Aorta Tissue

For histological analysis, mice were sacrificed by CO_2_ inhalation (number of mice used per analysis is indicated in the corresponding figure legends). Mice were perfused through the left ventricle with PBS. Aortas were fixed in formalin, segmented, and embedded in 3% Bacto agar. Embedded aortas were dehydrated through the histokinette processor (Microm) and subsequently paraffin embedded, after which 4 μm sections were prepared and placed on slides. Resorcin‐Fuchsin (RF) staining was performed to visualize the elastin structure of the vessel wall. Elastin fragmentation was counted manually and corrected for surface size. Alcian Blue (AB) staining was performed to visualize proteoglycan deposition in the aorta. A macro in Fiji (ImageJ) was designed to determine the mean percentage of AB positive area. In short, eosin counterstaining was removed from the image and an overlay mask was created to measure the staining intensity relative to the total section surface. The percentage of stained pixels was determined by setting a threshold to distinguish stained pixels from the background.

For immunohistochemical analysis, aorta sections were deparaffinized in xylene, rehydrated, and washed in washing buffer (PBS with 0.025% Triton X‐100). Antigen retrieval was performed by boiling in antigen retrieval buffers EDTA buffer (10 mM Tris base, 1 mM EDTA solution, pH 9.0) for MAC2 and citrate buffer (10 mM Sodium citrate, 0.05% Tween‐20, pH 6.0) for VCAM1 at 300 W for 15 min. Sections were treated with 3% H_2_O_2_ in methanol for 10 min to block endogenous peroxidase activity and subsequently blocked with 5% Protifar in washing buffer for 1 h. Sections were incubated overnight at 4°C with primary antibodies diluted in antibody buffer (1% Protifar in washing buffer) (Table S2). The following day, sections were washed with washing buffer and incubated with biotinylated secondary antibody diluted in antibody buffer (1:200, DAKO) for 30 min at room temperature (RT). Subsequently, sections were washed with washing buffer and incubated with avidin‐biotinylated complex diluted in washing buffer (Vectastain Universal Elite ABC kit Vector Laboratories) for 30 min at RT. After a final washing step, sections were incubated with DAB chromogen (DAKO Liquid Dab substrate‐chromogen system) as a substrate and subsequently incubated in CuSO_4_ for 5 min to boost the signal. Sections were counterstained with eosin. After dehydrating the sections and clearing them with xylene, they were mounted with Pertex mounting medium. Positive cells were counted manually and corrected for surface area.

### 2.11 Isolation of VSMCs From Mouse Aorta

*Ercc1* ^*Δ/−*^ (*n* = 3) and *Ercc1* ^*+/+*^ (*n* = 3) mice were sacrificed at 6 weeks of age and perfused through the left ventricle with PBS. Aortas were isolated and incubated in 2 mg/mL collagenase type II (LS004176, Worthington) at 37°C, 5% CO_2_ for 1–6 h until the tissue was dissociated. Primary VSMCs were centrifuged, resuspended in Dulbecco's Modified Eagle's Medium (DMEM) (Gibco) supplemented with 10% fetal calf serum (FCS) and 1% penicillin–streptomycin (PS) and cultured on dishes coated with 0.1% gelatin at 37°C, 5% CO_2_.

### 2.12 Immunofluorescent Staining of VSMCs

To investigate cGAS‐STING pathway activation in vitro, *Ercc1* ^*Δ/−*^ and *Ercc1* ^*+/+*^ VSMCs (passage numbers 9–12) were seeded in 12‐well plates (50,000 cells/well) on coverslips coated with 0.1% gelatin. The next day, cells were treated with 2 μg/mL cisplatin (Accord; infusion concentrate used in the clinic) for 48 h. After incubation, VSMCs were fixed with ice cold 100% methanol for 10 min and permeabilized with ice cold acetone for 1 min (for STING staining) or fixed with 2% paraformaldehyde in PBS for 15 min (for cGAS and ssDNA staining). Fixed cells were washed with PBS/0.1% Triton X‐100 and blocked with PBS+ (PBS supplemented with 0.5% bovine serum albumin (BSA) and 0.15% glycine) for 20 min. Next, cells were incubated overnight at 4°C with primary antibody diluted in PBS+ (Table S2). After washing, cells were incubated for 1 h at RT with secondary antibodies diluted in PBS+ (Table S2) followed by Hoechst 33342 staining (1:1000 in PBS, ThermoFisher). Images were recorded on a Stellaris 5 confocal microscope (Leica) with a 40x oil objective. Staining intensities of STING and cGAS in the cytoplasm were quantified using an in‐house developed macro in Fiji (ImageJ). Nuclei were selected based on Hoechst staining, and the mean intensity of STING or cGAS staining was measured outside the nuclei mask. The number of ssDNA spots was quantified by counting spots in Fiji and correcting for the number of nuclei. Experiments were performed in duplicates.

### 2.13 Immunoblot

For immunoblot analysis, mice were sacrificed at an age of 22 weeks (*n* = 3 per group) and perfused with PBS. Aortic tissue was isolated, snap frozen in liquid nitrogen, and homogenized in 1× Laemmli buffer (2% SDS, 10% glycerol, 60 mM Tris pH 6.8) using the TissueLyser 24 (Shanghai Jing Xin). Samples were heated for 10 min at 65°C and pulled through a 25G syringe to make them less viscous. The protein concentration was determined using the Lowry assay. Proteins (10 μg per sample) were separated by SDS‐PAGE and transferred to PVDF membranes (1.5 h, 100 V, 4°C, Immobilon). Total protein was measured using the Revert 700 Total Protein Stain kit (LI‐COR biosciences). Membranes were blocked for 1 h at RT with 3% milk in PBST (PBS + 0.1% Tween‐20) and incubated with primary antibodies overnight at 4°C (Table S2). After washing with PBST, membranes were incubated with horseradish peroxidase‐conjugated secondary antibodies (Table S2) for 1 h at RT. After washing, secondary antibodies were detected with the Amersham Imager 600 (GE Healthcare Life Sciences) using chemiluminescence. The intensity of the signal was measured using Fiji image analyzing software.

### 2.14 Statistics

Statistical analysis was performed using GraphPad Prism 10.2.0 (GraphPad Software Inc., La Jolla, California, USA). The D'Agostino‐Pearson (omnibus K2) test was used to test for normal distribution of the data. For normally distributed data, a two‐way ANOVA was applied to evaluate the effects of age and genotype or diet and genotype. For non‐normally distributed data, the Kruskal–Wallis test was used to determine significant differences between groups. The Sidak test was employed to correct for multiple comparisons. A *p*‐value < 0.05 was considered significant. Results are expressed as mean ± standard deviation (SD). In the figures, statistical significance is indicated as follows: one asterisk (*) indicates a *p*‐value < 0.05, two asterisks (**) indicate a *p*‐value < 0.01, three asterisks (***) indicate a *p*‐value < 0.001, and four asterisks (****) indicate a *p*‐value < 0.0001.

## 3 Results

### 3.1 Dietary Restriction Improves Left Ventricle Function in *Ercc1* ^*Δ/−*^ Mice

**FIGURE 1:**
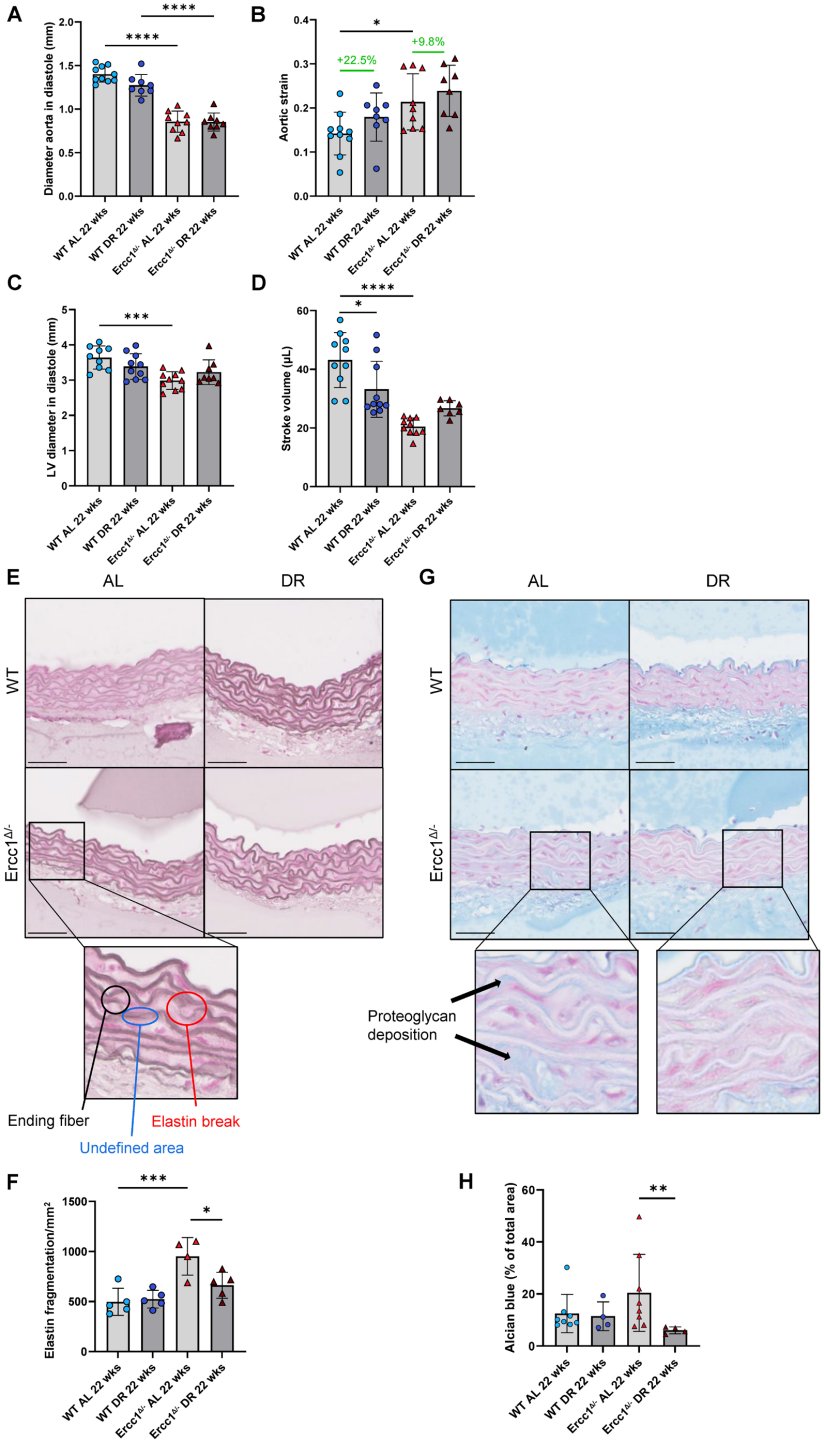
Dietary restriction improves left ventricle function and alleviates structural changes in Ercc1 mutant mouse aorta. (A) Aortic arch diameter in diastole and (B) aortic strain of *Ercc1* ^*Δ/−*^ and WT mice on AL or DR diet at 22 weeks of age. The average value of 3 individual measurements is plotted per mouse (mean ± SD, *n* = 8–10 per group, two‐way ANOVA, *p < 0.05, ****p < 0.0001). (C) Left ventricle diameter in diastole and (D) stroke volume of *Ercc1* ^*Δ/−*^ and WT mice on AL or DR diet at 22 weeks of age. The average value of 3 individual measurements is plotted per mouse (mean ± SD, *n* = 8–10 per group, two‐way ANOVA, *p < 0.05, ***p < 0.001, ****p < 0.0001). (E) Resorcin‐Fuchsin staining for elastin fibers identifying ending fibers, undefined areas and elastin breaks in the *Ercc1* ^*Δ/−*^ aorta. Scale bar = 50 μm (F) The total number of elastin fragmentations corrected for aortic media surface area was plotted (mean ± SD, *n* = 4–5 per group, two‐way ANOVA, *p < 0.05, ***p < 0.001). (G) Alcian Blue staining for proteoglycans identifying proteoglycan deposition in the *Ercc1* ^*Δ/−*^ aorta. *Scale bar = 50 μm* (H) The percentage of Alcian Blue positive area of the total aortic media surface area was plotted (mean ± SD, *n* = 4–8 per group, Kruskal–Wallis, **p < 0.01).

To investigate the aorta and left ventricle (LV) diameter and function in *Ercc1* ^*Δ/−*^ mice and WT littermates and the impact of DR on these parameters, we used ultrasound imaging. Measurements were performed at 22 weeks of age because all *Ercc1* ^*Δ/−*^ mice on an AL diet show advanced age‐related symptoms, including vascular aging, at this timepoint (van der Linden et al. 2024). Aorta diameter was significantly smaller in *Ercc1* ^*Δ/−*^ mice compared to WT littermates at 22 weeks of age, in both the AL‐fed and DR conditions (Figure 1A). Since *Ercc1* ^*Δ/−*^ mice experience growth retardation, they are significantly smaller in terms of total body size and bodyweight compared to WT littermates, and therefore aorta diameter is expected to be smaller (Figure S1) (Dollé et al. 2011; Waard et al. 2010). DR did not affect aorta diameter in either genotype. However, aortic strain, which measures the stiffness of the aorta, was significantly increased in AL‐fed *Ercc1* ^*Δ/−*^ mice compared to WT littermates (Figure 1B). It should be noted that alterations in blood pressure can influence these measurements, and *Ercc1* ^*Δ/−*^ mice were reported to have higher blood pressure compared to WT littermates (Durik et al. 2012). Although not significant, DR appeared to increase aortic strain, indicating a decreased stiffness in both genotypes (Figure 1B). Similarly, LV diameter in diastole was significantly smaller in AL‐fed *Ercc1* ^*Δ/−*^ mice compared to WT littermates (Figure 1C). Fractional shortening, a measure of LV contractility, and ejection fraction, a measure of LV pumping efficiency, were both significantly reduced in AL‐fed *Ercc1* ^*Δ/−*^ mice compared to WT littermates (Figure S2). Both parameters appeared to increase with DR in *Ercc1* ^*Δ/−*^ mice, although this effect did not reach statistical significance. Stroke volume, the volume of blood ejected from the LV per cardiac cycle, was significantly lower in AL‐fed *Ercc1* ^*Δ/−*^ mice compared to WT littermates. While not significant, DR tended to increase stroke volume in *Ercc1* ^*Δ/−*^ mice (Figure 1D). In WT mice, DR significantly reduced stroke volume, indicating that DR can have differential effects depending on genotype and health status.

Next, we examined whether these functional changes were related to structural changes in the aortic wall. Since vascular aging is characterized by elastin fragmentation, we performed a Resorcin‐Fuchsin staining to analyze elastin structure (Figure 1E). Elastin fragmentation was significantly increased in the *Ercc1* ^*Δ/−*^ aorta compared to the WT aorta of mice on an AL diet, consistent with previous reports (Figure 1F) (van der Linden et al. 2024). Notably, elastin fragmentation was significantly decreased in *Ercc1* ^*Δ/−*^ mice on DR compared to AL‐fed *Ercc1* ^*Δ/−*^ mice (Figure 1F). Another indication of extracellular matrix (ECM) remodeling is the deposition of proteoglycans, visualized by Alcian Blue staining. Alcian Blue staining was non‐significantly increased in the aorta of *Ercc1* ^*Δ/−*^ mice compared to the aorta of WT mice on an AL diet, consistent with previous findings (Figure 1G). Remarkably, DR significantly reduced Alcian Blue staining in the aortic wall of *Ercc1* ^*Δ/−*^ mice. Together, these results suggest that DR can partially reverse the structural and functional abnormalities observed in the aging aorta and heart of *Ercc1* ^*Δ/−*^ mice.

### 3.2 RNA Sequencing Analysis Confirms Previously Identified Aging Characteristics in the Aorta of *Ercc1* ^*Δ/−*^ Mice

**FIGURE 2:**
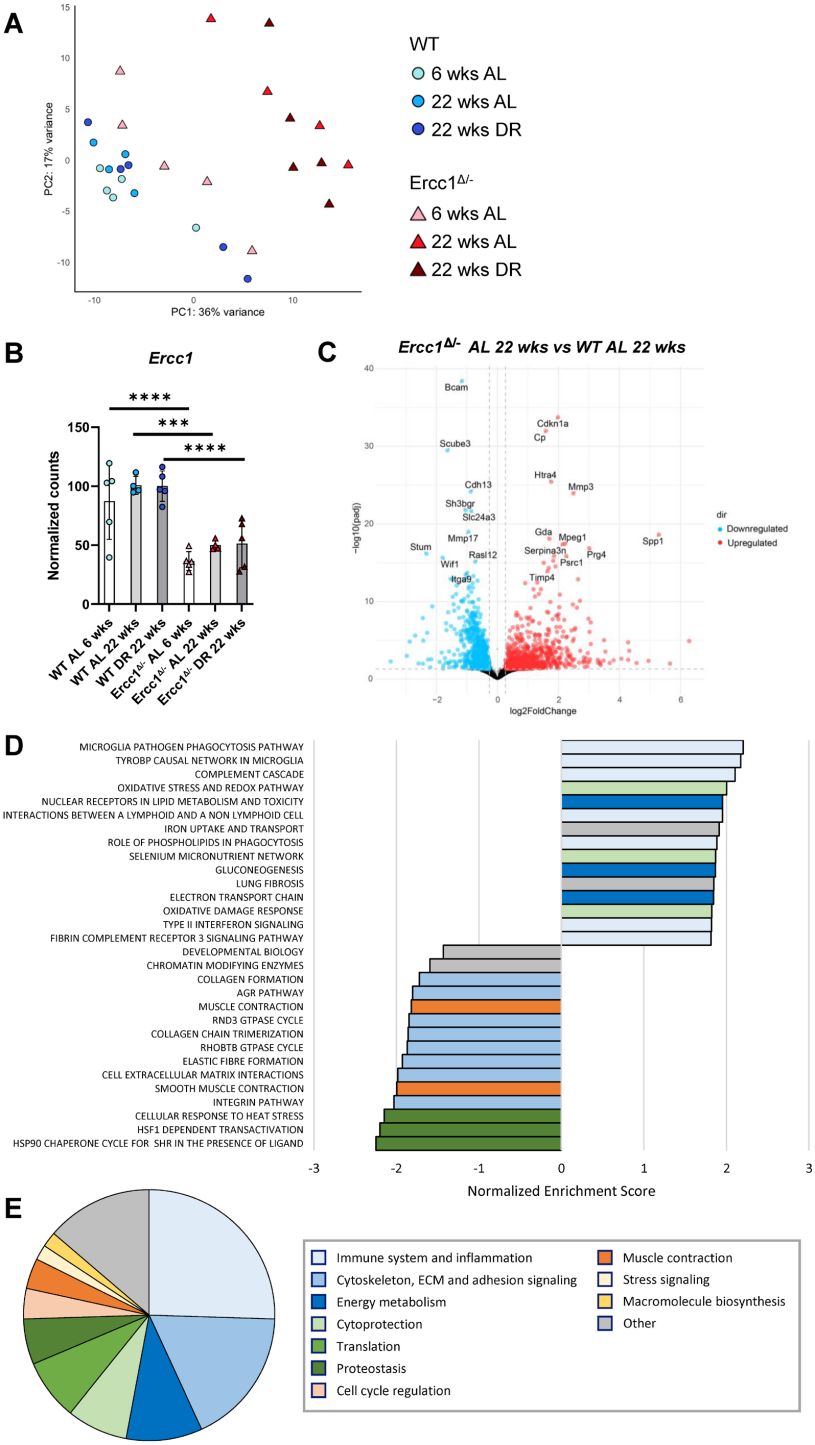
RNA sequencing analysis confirms previously identified aging characteristics in the aorta of Ercc1 mutant mice (A) PCA plot showing the similarity in RNA expression pattern between the different samples from the aortic arch of *Ercc1* ^*Δ/−*^ and WT mice. (B) Expression of the *Ercc1* gene (normalized counts) in the *Ercc1* ^*Δ/−*^ aortic arch compared to the WT aortic arch. The mean ± SD is plotted (*n* = 4–5 per group, DEG analysis using DESeq2 ***p < 0.001, ****p < 0.0001). (C) Volcano plot of genes differentially expressed between the 22‐week‐old *Ercc1* ^*Δ/−*^ aortic arch and the 22‐week‐old WT aortic arch of AL‐fed mice. The top 10 differentially expressed genes in each direction are highlighted (ranked based on significance, *Timp4* (16th most significantly upregulated gene) is additionally highlighted in the plot). Significantly upregulated genes are colored red and significantly downregulated genes are colored blue (adjusted *p*‐value ≤ 0.05, abs(log2FoldChange) ≥ 0.26 (log2(1.2)). (D) The top 15 most significantly enriched canonical pathways in each direction in the 22‐week‐old *Ercc1* ^*Δ/−*^ aortic arch compared to the WT aortic arch of AL‐fed mice. The bar coloring corresponds to the pathway categories described in the legend of figure E. (E) Pie chart showing the different categories of all significantly enriched canonical pathways.

To further explore vascular aging in *Ercc1* ^*Δ/−*^ mice, bulk mRNA sequencing was conducted on the aorta of *Ercc1* ^*Δ/−*^ and WT mice using the Illumina NovaSeq6000 platform. Differential expression analysis of the aortic arch of 6‐week‐old *Ercc1* ^*Δ/−*^ mice compared to their WT littermates revealed 373 (Table 1) significant differentially expressed genes (DEGs). At 22 weeks of age, differential expression analysis revealed 1719 significant DEGs between these two groups. This 4.6‐fold increase in the total number of DEGs between *Ercc1* ^*Δ/−*^ and WT mice in a time span of 18 weeks reflects the accelerated aging phenotype of *Ercc1* ^*Δ/−*^ mice. Principal Component Analysis (PCA) showed a full separation between *Ercc1* ^*Δ/−*^ and WT samples, indicating that genotype is the most prominent difference. Furthermore, samples of 6‐week‐old *Ercc1* ^*Δ/−*^ mice clustered more closely with those of WT mice than samples of 22‐week‐old *Ercc1* ^*Δ/−*^ mice did, indicating a progressive shift in RNA expression pattern upon aging (Figure 2A). Differential expression data confirmed significantly decreased *Ercc1* gene expression in the *Ercc1* ^*Δ/−*^ aorta compared to WT aorta (Figure 2B).

**TABLE 1:** The number of DEGs resulting from the different comparisons performed in DESeq2 on RNA sequencing data from Ercc1 and WT mouse aortas. Created in BioRender. Van der pluijm, I. (2025) https://BioRender.com/v52v937.

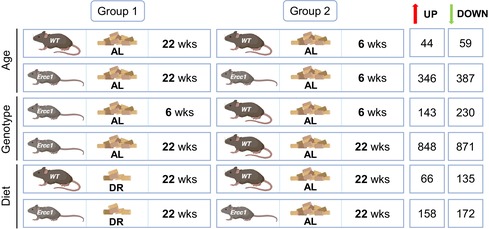

The top 20 most significantly changed genes in each direction in 22‐week‐old aortas from AL‐fed *Ercc1* ^*Δ/−*^ compared to AL‐fed WT mice contained ECM remodeling genes such as *Mmp3*, *Mmp17* and *Timp4* (Figure 2C). Additionally, the gene encoding stress/senescence marker p21 (*Cdkn1a*) was significantly upregulated, confirming prior findings of increased ECM remodeling and p21 expression in *Ercc1* ^*Δ/−*^ aortas (van der Linden et al. 2024). Gene Set Enrichment Analysis (GSEA) further indicated significant enrichment of pathways involved in ECM remodeling, including “*elastic fibre formation*”, “*collagen chain trimerization*” and *“collagen formation”* (Figure 2D). The “*muscle contraction*” pathway had a negative enrichment score, indicating downregulation of contraction‐related genes in the *Ercc1* ^*Δ/−*^ aorta. This was consistent with previous studies revealing decreased protein expression of contractile VSMC markers (van der Linden et al. 2024). Additionally, pathways related to oxidative stress and redox responses had a positive enrichment score, suggesting upregulation of these processes (Figure 2D). This was consistent with previous findings showing the increased accumulation of oxidative DNA damage and elevated reactive oxygen species (ROS) levels in *Ercc1* ^*Δ/−*^ liver and kidney (Robinson et al. 2018). Further analysis with Ingenuity Pathway Analysis (IPA) revealed downregulation of transcription factors *Jun* and *Srf*, which are downstream of the IGF‐1 signaling pathway, and predicted inhibition of this pathway in the 22‐week‐old *Ercc1* ^*Δ/−*^ aorta of AL‐fed mice (Figure S3). Furthermore, GSEA analysis highlighted immune response pathways including “*microglia pathogen phagocytosis pathway*”, “*complement cascade*” and “*type II interferon signaling*”, and categorization of enriched pathways revealed that “*immune system and inflammation*” was the largest category containing 14 of 61 pathways (Figure 2E). These findings suggest that Ercc1 deficiency triggers a pro‐inflammatory immune response in the aorta, similar to effects seen in other tissues such as adipose tissue, the kidney and the pancreas of *Ercc1* ^*Δ/−*^ mice (Karakasilioti et al. 2013; Chatzidoukaki et al. 2021; Schermer et al. 2013). Altogether, these findings revealed that mechanisms previously observed in *Ercc1* ^*Δ/−*^ mice in different tissues were also visible at the RNA expression level in the aorta of AL‐fed *Ercc1* ^*Δ/−*^ mice.

### 3.3 Dietary Restriction Improves Oxidative Damage Response, Immune Response, and Metabolism in the Aorta of Ercc1^Δ^ ^/−^ Mice

**FIGURE 3:**
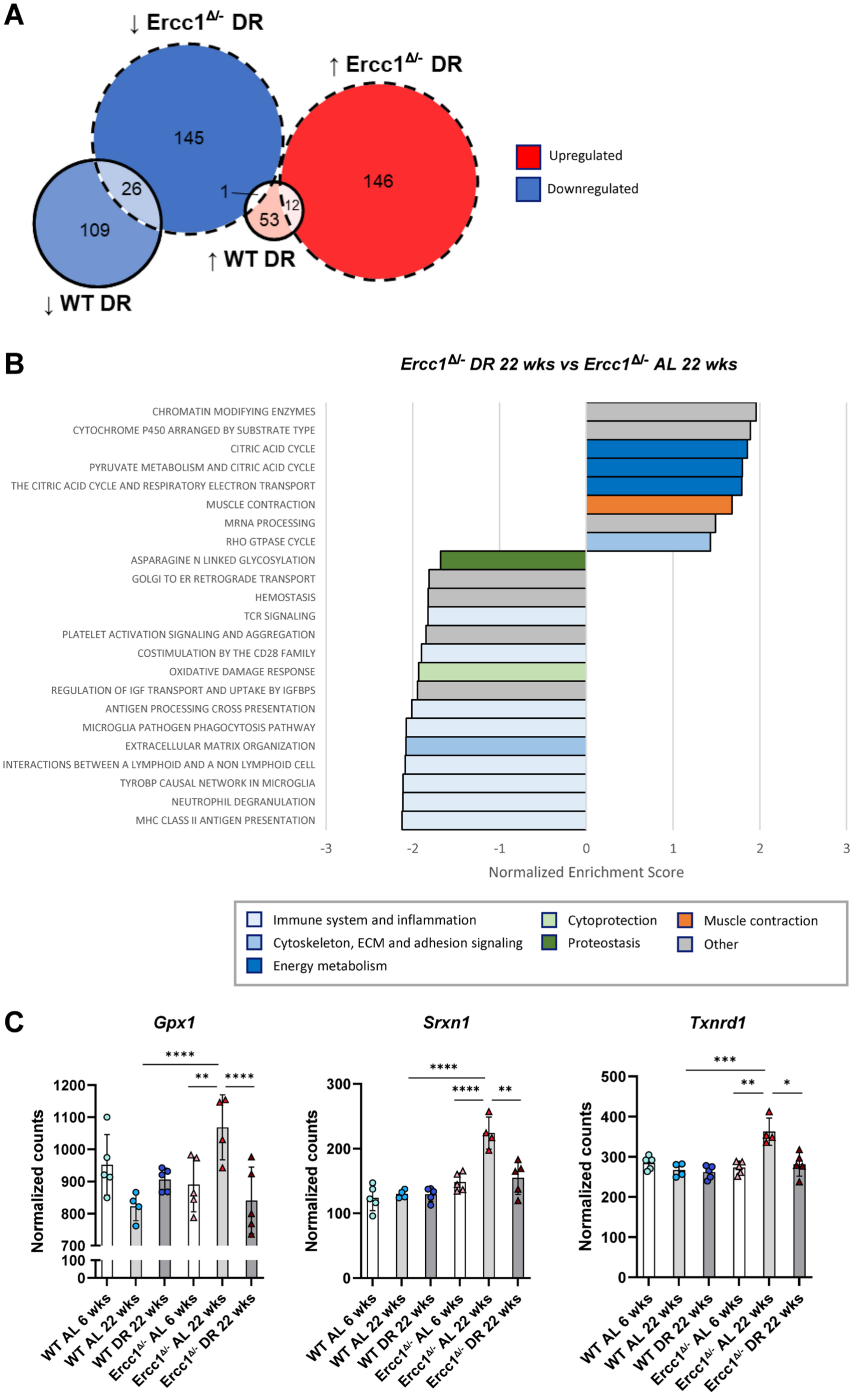
Dietary restriction affects the oxidative damage response, immune response and metabolism in the aorta of Ercc1 mutant mice. (A) Euler diagram showing the overlap between genes that are significantly differentially expressed in the *Ercc1* ^*Δ/−*^ aorta as an effect of DR (numbers within circle with dotted line) and genes that are significantly differentially expressed in the WT aorta as an effect of DR (numbers within circle with solid line). (B) The significantly enriched canonical pathways in the aortic arch of *Ercc1* ^*Δ/−*^ mice on DR compared to the aortic arch of AL‐fed *Ercc1* ^*Δ/−*^ mice. The bar coloring corresponds to the pathway categories described in the legend. (C) RNA expression of the genes *Gpx1*, *Sxrn1* and *Txnrd1* that encode antioxidant proteins. The normalized counts (mean ± SD) are plotted (*n* = 4–5 per group, *p < 0.05, **p < 0.01, ***p < 0.001, ****p < 0.0001).

To assess the effect of DR on vascular aging, we compared RNA expression in the aorta of DR‐fed 22‐week‐old mice to AL‐fed 22‐week‐old mice. Differential expression analysis revealed 330 significant DEGs in the aortic arch of *Ercc1* ^*Δ/−*^ mice on DR compared to the same group on an AL diet (Table 1). In WT mice, 201 DEGs were identified when comparing mice on DR to AL‐fed mice. When matching these two gene sets, only 38 genes were found to have a shared response upon DR in *Ercc1* ^*Δ/−*^ mice and WT mice (26 downregulated genes, 12 upregulated genes), with 1 DEG switching direction (Figure 3A). This suggests that DR affects *Ercc1* ^*Δ/−*^ mice at least in part differently than WT mice. GSEA analysis revealed that DR downregulated the upregulated “*oxidative damage response”* pathway in *Ercc1* ^*Δ/−*^ mice (Figure 3B). Specifically, antioxidant genes *Gpx1*, *Srxn1* and *Txnrd1* were upregulated in the 22‐week‐old *Ercc1* ^*Δ/−*^ aorta compared to WT aorta and DR restored their expression to baseline levels in *Ercc1* ^*Δ/−*^ mice (Figure 3C). Additionally, immune response pathways like “*MHC class II antigen presentation*” and “*microglia pathogen phagocytosis pathway*” were downregulated by DR (Figure 3B). Furthermore, pathways involved in energy metabolism, such as “*the citric acid cycle and respiratory electron transport*”, were altered by DR. These results suggest that DR mitigates processes that contribute to impaired structure and function in the aging *Ercc1* ^*Δ/−*^ aorta.

**FIGURE 4:**
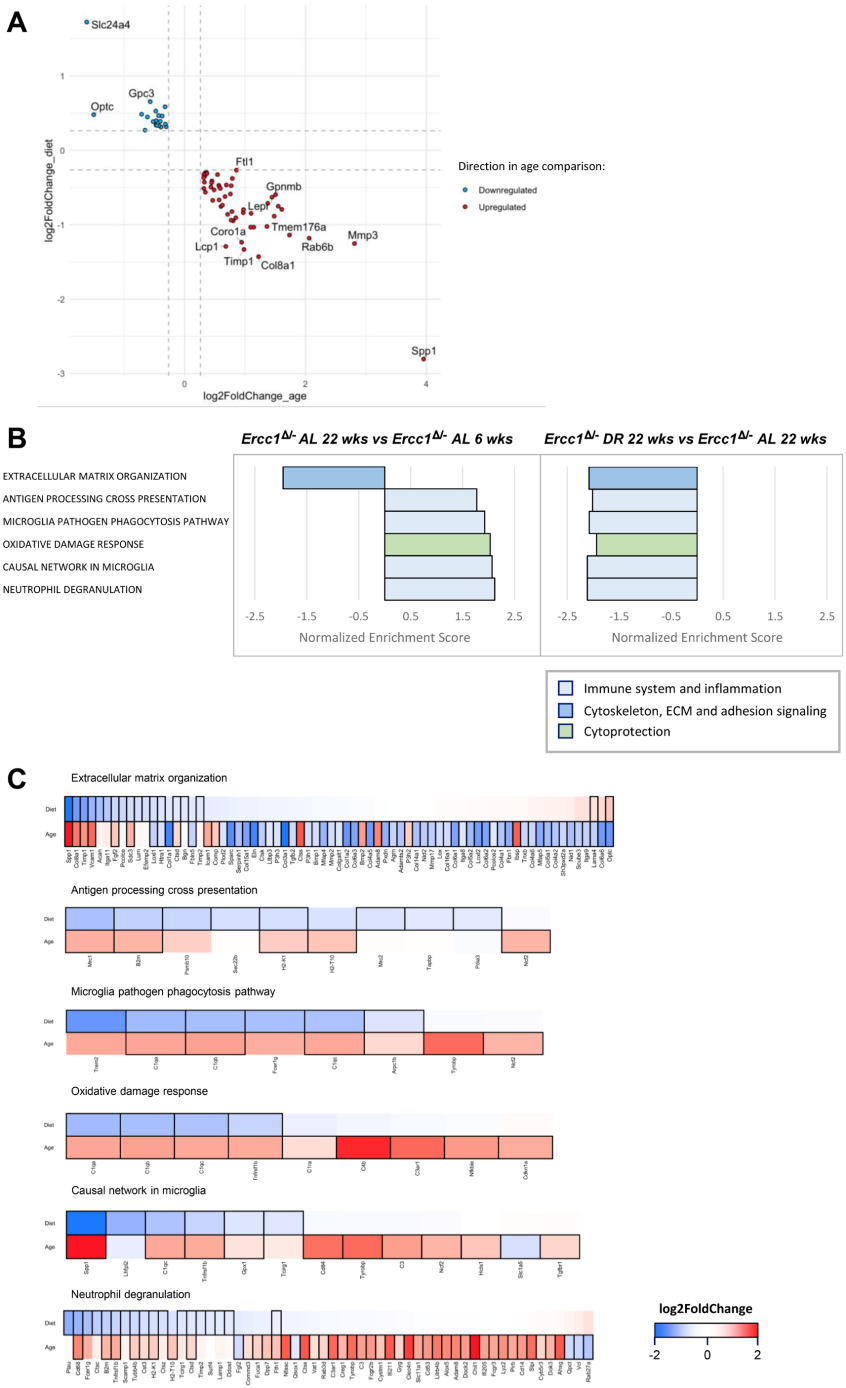
Genes that switch direction upon dietary restriction in the Ercc1 mutant mouse aorta. (A) Plot showing the log2FoldChange of genes that are significantly differentially expressed in the ‘age’ comparison (*x*‐axis, 22‐week‐old vs. 6‐week‐old AL‐fed *Ercc1* ^*Δ/−*^ aorta) and that switch direction (from upregulated to downregulated expression or vice versa) upon DR (y‐axis, DR vs. AL‐fed *Ercc1* ^*Δ/−*^ aorta). Genes that are significantly upregulated in the ‘age’ comparison are colored red and significantly downregulated genes are colored blue (adjusted *p*‐value ≤ 0.05, abs(log2FoldChange) ≥ 0.26 (log2(1.2))). (B) Canonical pathways detected by GSEA that are significantly enriched based on the set of switching genes presented in (A). (C) The genes that are changed in the canonical pathways resulting from the GSEA analyses presented in (B). The outlined squares are significant in that comparison (adjusted *p*‐value ≤ 0.05).

To explore the effects of DR on accelerated aging in *Ercc1* ^*Δ/−*^ mice, we focused on significant DEGs in aging (22 weeks vs. 6 weeks, AL) that were also changed upon DR (DR vs. AL, 22 weeks) but switched directions (upregulated in aging but downregulated by DR, and vice‐versa). Among the 71 genes that switched direction, 52 were upregulated in aging but downregulated by DR, while 19 were downregulated in aging but upregulated by DR (Figure 4A). GSEA pathway analysis showed enrichment in pathways involved in ECM remodeling, oxidative damage response, and immune response (Figure 4B,C) further supporting that DR alleviates these processes in the *Ercc1* ^*Δ/−*^ aorta.

### 3.4 Dietary Restriction Reduces ECM Remodeling and MMP Activity in the Aorta of Ercc1^Δ^ ^/−^ Mice

**FIGURE 5:**
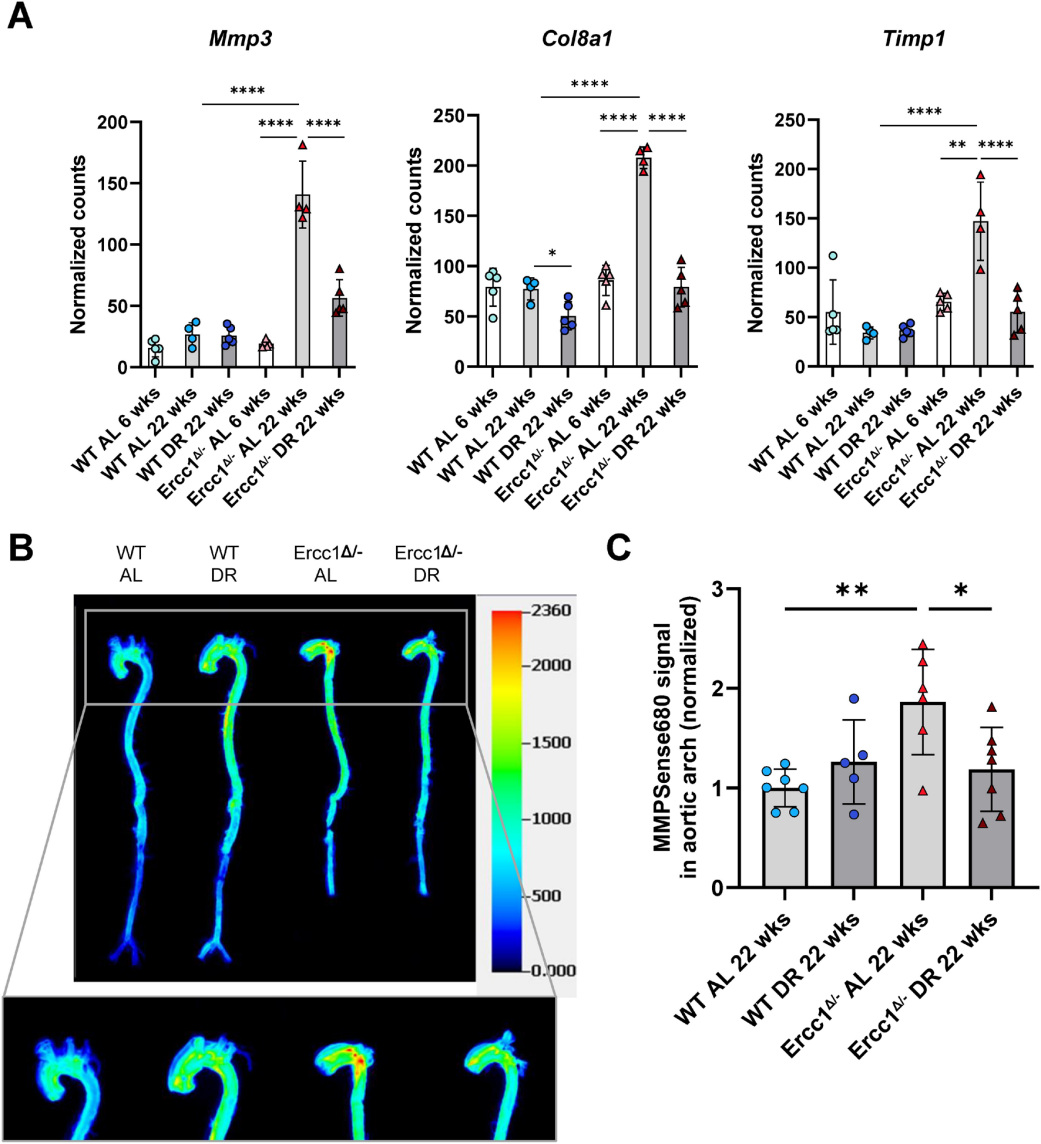
Dietary restriction alleviates ECM remodeling in the Ercc1 mutant mouse aorta. (A) RNA expression of the genes *Mmp3*, *Col8a1* and *Timp1* that encode proteins involved in ECM remodeling. The normalized counts (mean ± SD) are plotted (*n* = 4–5 per group, DEG analysis using DESeq2 *p < 0.05, **p < 0.01, ****p < 0.0001). (B) Representative ex vivo image of aortas from *Ercc1* ^*Δ/−*^ and WT mice on DR or AL diet, injected with MMPSense680. (C) The fluorescent signal indicative of MMP activity was measured in the aortic arch and normalized toward the signal from the WT AL samples in each individual group. The normalized fluorescent signal is plotted per mouse (mean ± SD, *n* = 5–7 per group, two‐way ANOVA, *p < 0.05, **p < 0.01).

RNAseq data suggested increased ECM remodeling in the aging *Ercc1* ^*Δ/−*^ aorta, which is alleviated upon DR, underlined by the expression of *Mmp3*, *Col8a1*, and *Timp1* (Figure 5A). These genes were upregulated in the aging *Ercc1* ^*Δ/−*^ aorta and downregulated by DR. Immunoblot analysis showed that MMP3 protein levels were not elevated in AL‐fed *Ercc1* ^*Δ/−*^ mice (Figure S4). Furthermore, a trend of increased TIMP1 protein expression was observed in the *Ercc1* ^*Δ/−*^ aorta of AL‐fed mice, which seemed to be non‐significantly decreased by DR (Figure S4). The discrepancy between RNA and protein levels of these markers could be attributed to post‐transcriptional regulation, which is tightly controlled given the critical role of MMPs in physiological processes. To assess functionally relevant changes, we used the protease‐activatable MMPSense680 probe to measure enzymatic activity of MMPs. MMP activity was significantly higher in the *Ercc1* ^*Δ/−*^ aorta compared to WT, and DR significantly reduced this activity (Figure 5B,C). Furthermore, DR significantly decreased fragmentation of elastin fibers, which is one of the substrates of MMP12, in the *Ercc1* ^*Δ/−*^ aorta (Figure 1H). These results suggest that DR reduces ECM remodeling in the *Ercc1* ^*Δ/−*^ aorta.

### 3.5 Dietary Restriction Attenuates Immune Activation and VSMC Phenotype Switching in the Aorta of Ercc1^Δ^ ^/−^ Mice

As mentioned above, our RNAseq data suggests the presence of an immune response in the aging *Ercc1* ^*Δ/−*^ aorta, which is downregulated by DR. To further investigate this, an upstream regulator (UR) analysis was performed using IPA, which predicts whether a protein, gene, drug, or chemical is activated or inhibited based on the differential expression of downstream genes. For drugs or chemicals, which are not endogenously present, IPA bases its findings on the expression of genes that are known to be altered by the drug or chemical from experimental findings. This analysis revealed that immunostimulatory agents, including poly rl:rC‐RNA and lipopolysaccharide, were predicted to be activated in the 22‐week‐old *Ercc1* ^*Δ/−*^ aorta compared to both the 6‐week‐old *Ercc1* ^*Δ/−*^ aorta and 22‐week‐old WT aorta (Figure S5). Conversely, these URs were predicted to be inhibited by DR specifically in the *Ercc1* ^*Δ/−*^ aorta, suggesting the presence of an immune response in the *Ercc1* ^*Δ/−*^ mouse aorta that may be alleviated by DR.

**FIGURE 6:**
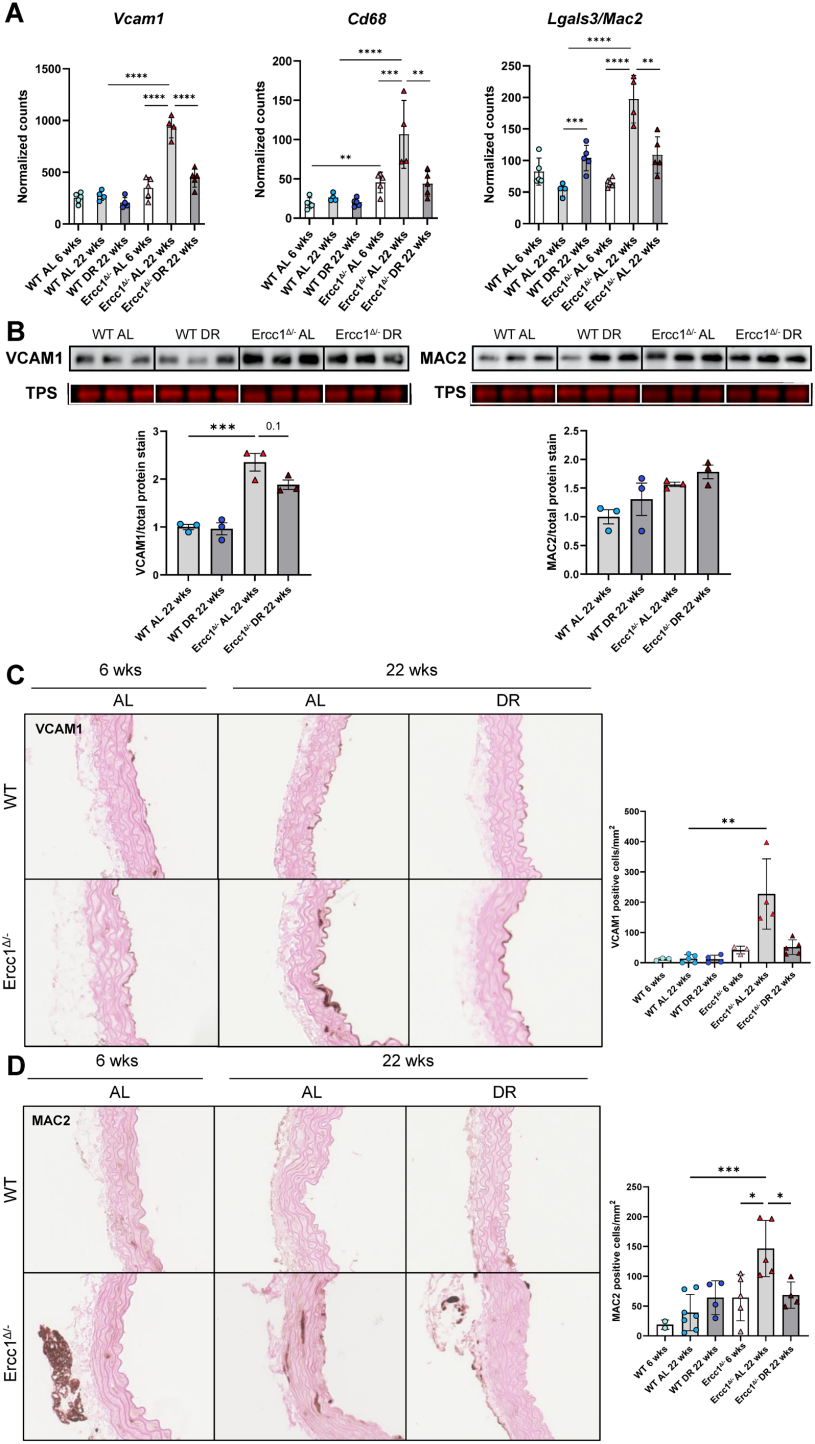
The presence of macrophage‐like VSMCs in the Ercc1 mutant mouse aorta is reduced by dietary restriction. (A) RNA expression of the genes *Vcam1*, *Cd68* and *Lgals3/Mac2* that encode markers for the macrophage‐like VSMC phenotype. The normalized counts (mean ± SD) are plotted (*n* = 4–5 per group, DEG analysis using DESeq2 **p < 0.01, ***p < 0.001, ****p < 0.0001). (B) Immunoblot showing VCAM1 and MAC2 protein expression in *Ercc1* ^*Δ/−*^ and WT mouse aorta. VCAM1 and MAC2 protein levels were corrected for total protein (Total Protein Stain). The average of 2 separate Western blots was plotted per mouse (mean ± SD, *n* = 3 per group, two‐way ANOVA, ***p < 0.001). (C) Representative images (left) and quantification (right) of immunohistochemical staining for VCAM1 on *Ercc1* ^*Δ/−*^ and WT mouse aortas. The number of VCAM1 positive cells corrected for aortic media surface area was plotted (mean ± SD, *n* = 3–5 per group, Kruskal–Wallis, **p < 0.01). (D) Representative images (left) and quantification (right) of immunohistochemical staining for MAC2 on *Ercc1* ^*Δ/−*^ and WT mouse aortas. The number of MAC2 positive cells corrected for aortic media surface area was plotted (mean ± SD, *n* = 2–6 per group, two‐way ANOVA, *p < 0.05, ***p < 0.001).

In response to stress or damage, contractile VSMCs can dedifferentiate and adapt their phenotype to address stressful conditions, such as high blood pressure or damage. One such phenotype is the macrophage‐like phenotype (Cao et al. 2022). These VSMCs acquire phagocytic properties and interact with both resident and non‐resident immune cells, which can trigger a chronic inflammatory state in the aortic wall and promote vascular aging (Sorokin et al. 2020). This macrophage‐like phenotype is characterized by the expression of marker genes *Vcam1*, *Cd68*, and *Lgals3/Mac2*. All three genes were upregulated in the *Ercc1* ^*Δ/−*^ aorta when compared between 22 and 6 weeks, as well as when compared to the WT aorta at 22 weeks (Figure 6A). Conversely, DR downregulated the expression of these genes in the *Ercc1* ^*Δ/−*^ aorta (Figure 6A). Immunoblot analysis confirmed increased expression of VCAM1 protein in the *Ercc1* ^*Δ/−*^ aorta compared to the WT aorta in AL‐fed mice (Figure 6B). DR resulted in a non‐significant trend toward decreased VCAM1 protein expression in the *Ercc1* ^*Δ/−*^ aorta (*p* = 0.1) (Figure 6B). Unlike our RNAseq findings, no decrease in MAC2 protein expression was observed upon DR in the *Ercc1* ^*Δ/−*^ aorta. To assess localization, immunohistochemical staining for VCAM1 and MAC2 was performed to identify cells positive for these markers (Figure 6C,D). Quantification of these stainings revealed a significantly increased number of VCAM1‐ and MAC2‐positive cells in the medial layer of the 22‐week‐old *Ercc1* ^*Δ/−*^ aorta compared to the 22‐week‐old WT aorta in AL‐fed mice (Figure 6C,D). DR significantly reduced the number of MAC2‐positive cells in the *Ercc1* ^*Δ/−*^ aorta and, although not significant, also reduced the number of VCAM1‐positive cells. Staining for CD3, a marker for T‐cells, confirmed that these were not immune cells infiltrating the aortic wall (Figure S6). Collectively, these results indicate that DR reduces the phenotypic switch of VSMCs to a macrophage‐like phenotype in the *Ercc1* ^*Δ/−*^ aorta.

### 3.6 Activation of the cGAS‐STING Pathway by DNA Damage in Ercc1^Δ^ ^/−^ Aorta Is Mitigated by Dietary Restriction

**FIGURE 7:**
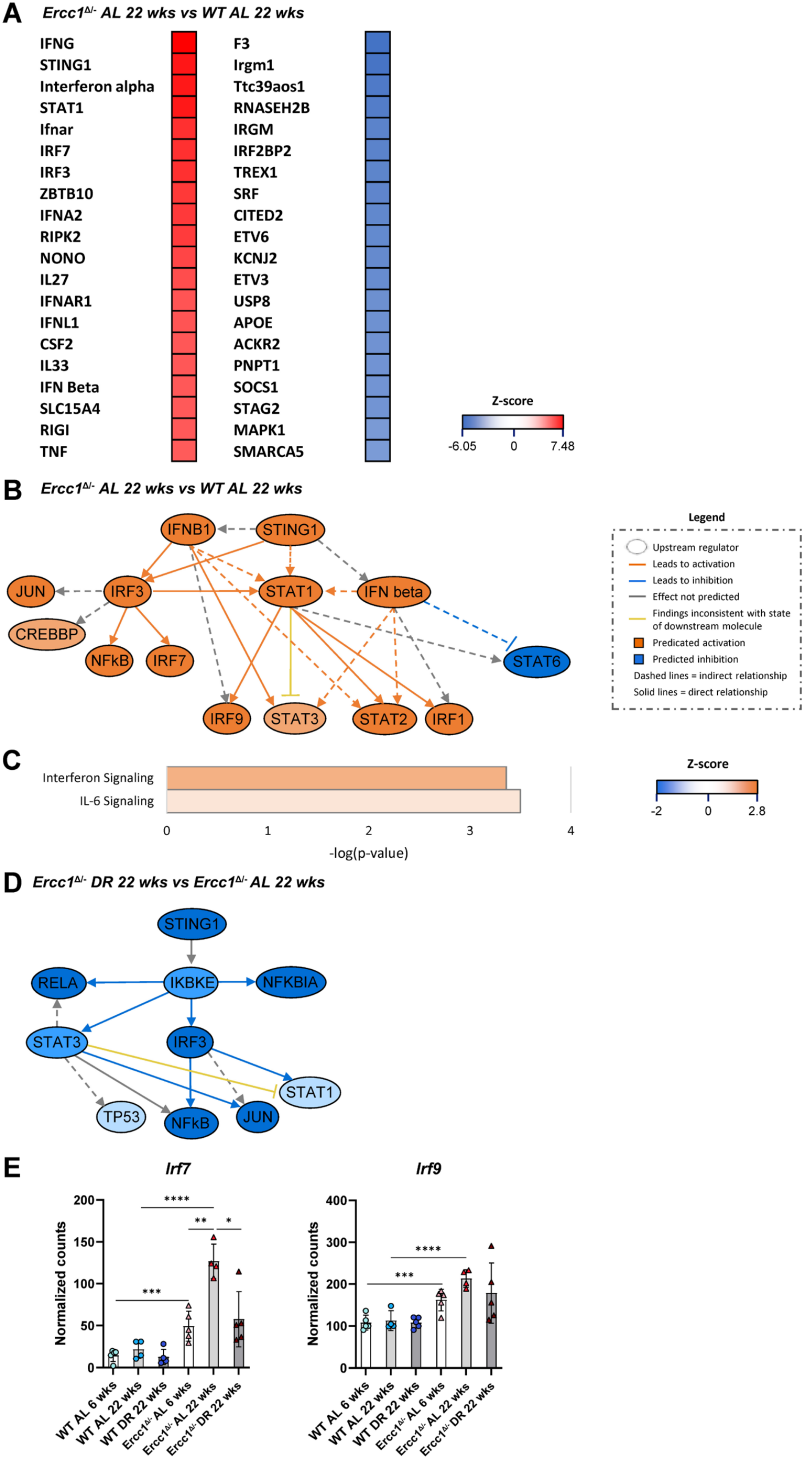
RNAseq data suggests activation of the cGAS‐STING pathway in the Ercc1 mutant mouse aorta and inhibition of this pathway by dietary restriction. (A) The top 20 activated and top 20 inhibited upstream regulators (proteins and genes) in the 22‐week‐old *Ercc1* ^*Δ/−*^ mouse aorta compared to WT aorta of AL‐fed mice. (B) Mechanistic network of upstream regulator STING1 based on the DEGs in the 22‐week‐old *Ercc1* ^*Δ/−*^ mouse aorta compared to WT aorta of AL‐fed mice. (C) IPA analysis of the canonical pathways *interferon signaling* and *IL‐6 signaling* in the 22‐week‐old *Ercc1* ^*Δ/−*^ mouse aorta compared to WT aorta of AL‐fed mice. (D) Mechanistic network of upstream regulator STING1 based on the DEGs in the *Ercc1* ^*Δ/−*^ aorta of mice on DR compared to mice on AL diet. (E) RNA expression of the genes *Irf7* and *Irf9* that encode transcription factors downstream of the cGAS‐STING pathway. The normalized counts (mean ± SD) are plotted (*n* = 4–5 per group, *p < 0.05, **p < 0.01, ***p < 0.001, ****p < 0.0001).

**FIGURE 8:**
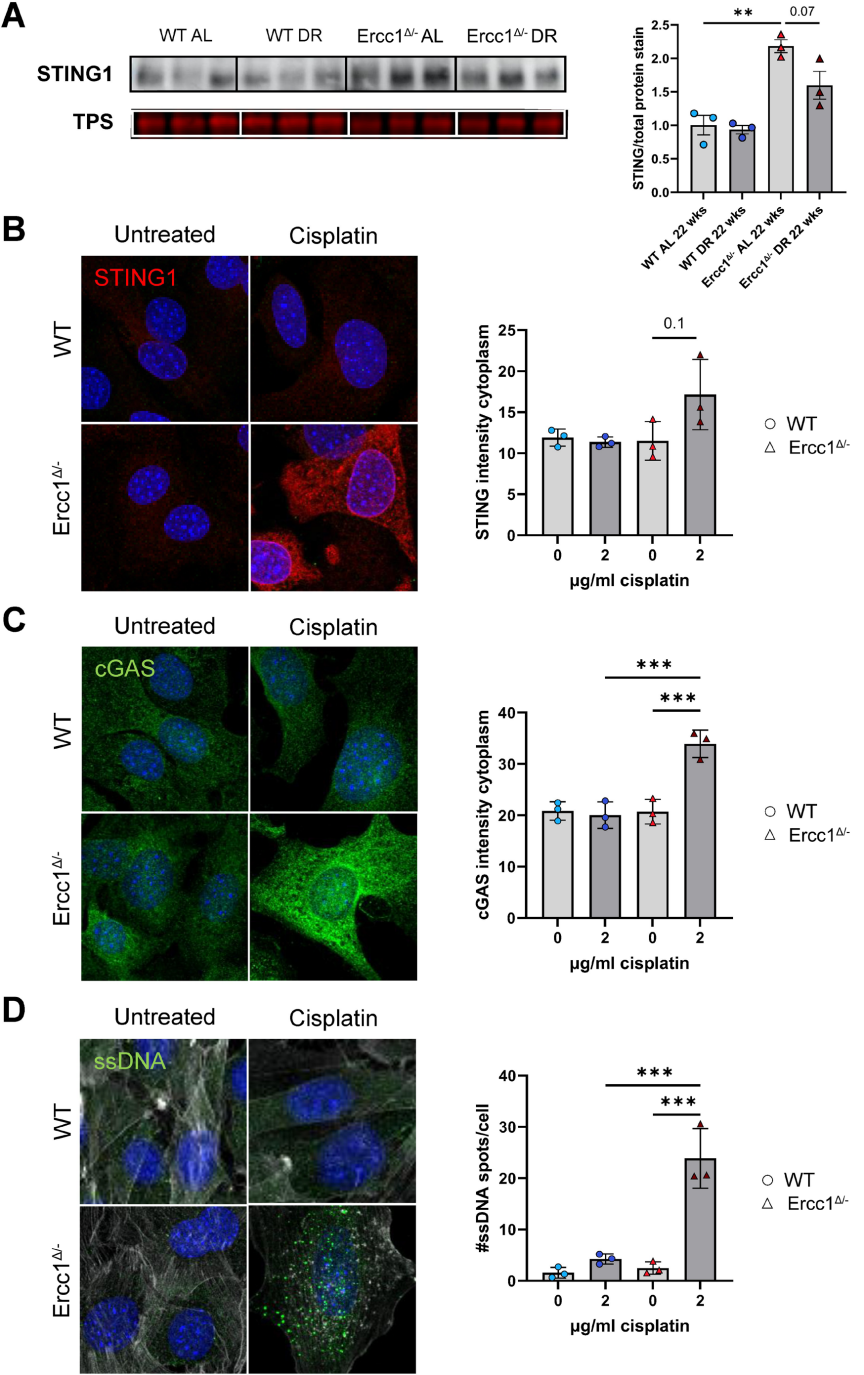
Increased expression of STING1 in the Ercc1 mutant mouse aorta and cisplatin‐induced activation of cGAS‐STING in Ercc1 mutant VSMCs. (A) Immunoblot showing STING1 protein expression in *Ercc1* ^*Δ/−*^ and WT mouse aorta. STING1 protein levels were corrected for total protein (Total Protein Stain). The average of 2 separate Western blots was plotted per mouse (mean ± SD, *n* = 3 per group, two‐way ANOVA, **p < 0.01). (B) Representative images (left) and quantification (right) of immunofluorescent staining for STING1 protein on *Ercc1* ^*Δ/−*^ and WT VSMCs with 0 or 2 μg/mL cisplatin treatment for 48 h. The average value of the STING1 intensity in the cytoplasm was plotted per cell line (mean ± SD, *n* = 3 per group, two‐way ANOVA). (C) Representative images (left) and quantification (right) of immunofluorescent staining for cGAS protein on *Ercc1* ^*Δ/−*^ and WT VSMCs with 0 or 2 μg/mL cisplatin treatment for 48 h. The average value of the cGAS intensity in the cytoplasm was plotted per cell line (mean ± SD, *n* = 3 per group, two‐way ANOVA, ***p < 0.001). (D) Representative images (left) and quantification (right) of immunofluorescent staining for ssDNA on *Ercc1* ^*Δ/−*^ and WT VSMCs with 0 or 2 μg/mL cisplatin treatment for 48 h. The average number of ssDNA spots per cell was plotted per cell line (mean ± SD, *n* = 3 per group, two‐way ANOVA, ***p < 0.001).

Defects in DNA damage repair are frequently linked to activation of the cGAS‐STING pathway (Chatzidoukaki et al. 2021). This activation, in turn, causes downstream expression of type I interferons and pro‐inflammatory cytokines including IL‐6 and TNF, which can trigger a DNA‐driven immune response. We therefore investigated whether activation of the cGAS‐STING pathway could underlie the observed immune response in the *Ercc1* ^*Δ/−*^ aorta. UR analysis revealed predicted activation of a number of proteins involved in the cGAS‐STING pathway in the 22‐week‐old *Ercc1* ^*Δ/−*^ aorta compared to the WT aorta in AL‐fed mice, such as interferon alpha, interferon beta, STING1, IRF7, and IRF3 (Figure 7A). Since STING1 is a key signal transducer in this pathway, we focused on its role. IPA generated mechanistic networks that connected URs to visualize how they might collaborate to induce the observed gene expression changes in our dataset. The mechanistic network for STING1 showed connections to several URs involved in the cGAS‐STING pathway, such as interferon beta, IRF3, IRF7, and NF‐κB, all of which were predicted to be activated (Figure 7B). Moreover, the canonical pathways downstream of cGAS‐STING, “*interferon signaling*” and “*IL‐6 signaling*” were both predicted to be activated (Figure 7C). Conversely, STING1 was predicted to be inhibited by DR in the aorta of *Ercc1* ^*Δ/−*^ mice (Figure 7D). The resulting mechanistic network again contained URs involved in the cGAS‐STING pathway such as IRF3 and NF‐κB, but in this comparison, they were predicted to be inhibited. Specifically, expression of the transcription factors *Irf7* and *Irf9*, which induce downstream expression of type I interferon genes, was significantly increased in the 22‐week‐old *Ercc1* ^*Δ/−*^ aorta compared to the WT aorta in AL‐fed mice (Figure 7E). DR reduced expression of these genes in the *Ercc1* ^*Δ/−*^ aorta, which was significant for *Irf7*. These findings suggest that the cGAS‐STING pathway is activated in the 22‐week‐old *Ercc1* ^*Δ/−*^ aorta and that DR inhibits this pathway. Immunoblot analysis confirmed significantly increased expression of STING1 protein in the 22‐week‐old *Ercc1* ^*Δ/−*^ mouse aorta compared to the WT aorta, with a trend toward decreased STING1 protein in the *Ercc1* ^*Δ/−*^ aorta following DR (*p* = 0.07) (Figure 8A).

Next, we validated in vitro that impaired DNA damage repair could cause activation of the cGAS‐STING pathway. UR analysis focused on drugs and chemicals and identified gene expression changes typically induced by cisplatin, a drug that causes DNA damage by forming intrastrand and interstrand DNA cross‐links, in the 22‐week‐old *Ercc1* ^*Δ/−*^ aorta of AL‐fed mice (Rocha et al. 2018) (Figure S5). These gene expression changes were reversed by DR in the *Ercc1* ^*Δ/−*^ aorta (Figure S5). The DNA damage induced by cisplatin is typically repaired by nucleotide excision repair and interstrand crosslink repair (Rocha et al. 2018), which are impaired in *Ercc1* ^*Δ/−*^ mice. These results suggest that DNA damage accumulation in the *Ercc1* ^*Δ/−*^ aorta with aging may cause cGAS‐STING activation, which can be reduced by DR. Therefore, we investigated whether cisplatin‐induced DNA damage could activate the cGAS‐STING pathway in *Ercc1* ^*Δ/−*^ VSMCs in vitro. After isolating VSMCs from mouse aorta, immunohistochemical staining for MYH11 and αSMA confirmed the VSMC phenotype (Figure S7). Treatment of VSMCs with 2 μg/mL cisplatin for 48 h resulted in a trend toward increased STING1 staining intensity (*p* = 0.1) in *Ercc1* ^*Δ/−*^ VSMCs, but not in WT VSMCs (Figure 8B). Consistently, cisplatin treatment induced a significant increase in cGAS staining intensity in the cytoplasm of *Ercc1* ^*Δ/−*^ VSMCs, but not in WT VSMCs (Figure 8C). Furthermore, cisplatin treatment induced the release of single‐stranded DNA (ssDNA) fragments into the cytoplasm of *Ercc1* ^*Δ/−*^ VSMCs (Figure 8D). Quantification showed a significant increase in the average number of ssDNA spots per cell following cisplatin treatment in *Ercc1* ^*Δ/−*^ VSMCs, but not in WT VSMCs (Figure 8D). These results suggest that cisplatin‐induced DNA damage, which cannot be adequately repaired by ERCC1‐deficient VSMCs, leads to the release of ssDNA fragments into the cytoplasm, which subsequently activates the cGAS‐STING pathway.

### 3.7 Upstream Regulator Analysis Identifies Potential DR Mimetics

Despite its therapeutic potential, DR has several limitations that make its clinical application challenging, including difficulties with adherence, potential impacts on quality of life, and adverse effects such as reduced bone and muscle mass. Therefore, our objective was to identify compounds that could mimic the beneficial effects of DR. To achieve this, we conducted an UR analysis focused on drugs and chemicals predicted to be activated by DR in the *Ercc1* ^*Δ/−*^ aorta. This analysis is based on the differential expression of genes in our dataset that, according to the literature, are affected by these compounds. Interestingly, this revealed predicted activation of sirolimus (rapamycin) and resveratrol, which are both known DR mimetics (Figure S8). Rapamycin is an inhibitor of the mTOR pathway and increases maximum lifespan in invertebrates and mice. More specifically, rapamycin treatment reverses age‐related vascular dysfunction and oxidative stress in naturally aged mice (Lesniewski et al. 2017). Resveratrol is a polyphenolic compound and has potential antioxidant and anti‐inflammatory activity. Resveratrol supplementation reduces atherosclerotic lesion size and promotes lipid metabolism in *ApoE* ^*−/−*^ mice (Cheng et al. 2023). These previous findings suggested that these compounds, at least in part, mimic the beneficial effects of DR and have the potential to alleviate vascular aging. Furthermore, losartan, an Angiotensin II type 1 receptor inhibitor, was predicted to be activated. Inhibition of the renin‐angiotensin system increases survival and prevents age‐associated diseases in mice (de Cavanagh et al. 2011). Interestingly, losartan has already shown potential as a therapy for vascular aging in the clinic as it reduces aortic stiffness in patients with age‐related hypertension (Zhang et al. 2018). UR analysis also showed predicted activation of two peroxisome proliferator‐activated receptor (PPAR) agonists: pirinixic acid and troglitazone. Through their anti‐inflammatory, anti‐atherogenic, and antioxidant effects, activation of PPARs protects against the vascular complications of hypertension, atherosclerosis, and myocardial infarction in animal models of metabolic diseases (Cheang et al. 2015). Thus, UR analysis identified compounds that may mimic the therapeutic effect of DR in the *Ercc1* ^*Δ/−*^ aorta, as suggested by previous studies in animal models and patients. Notably, the analysis also predicted activation of the MAC2 inhibitor TD139, which is relevant given that MAC2 is a marker for the macrophage‐like VSMC phenotype, and our results suggested that DR reversed the switch to this phenotype in the *Ercc1* ^*Δ/−*^ aorta. Thus, this UR analysis identified several potential pharmacological alternatives to DR that could mitigate vascular aging.

## 4 Discussion

Aging is a major risk factor in the development of CVDs, which are currently the leading cause of death globally. Therefore, there is a need for improved therapeutic interventions that could delay or possibly prevent cardiovascular aging. In this study, we aimed to identify the molecular mechanisms that underlie cardiovascular aging in DNA repair deficient *Ercc1* ^*Δ/−*^ mice as well as factors that drive the possible therapeutic effect of DR in the aorta. To gain more insight into these mechanisms, we performed bulk mRNA sequencing on aortas of *Ercc1* ^*Δ/−*^ mice that had either been subjected to an AL diet or 30% DR.

RNA sequencing data of the *Ercc1* ^*Δ/−*^ aorta reflected previous findings in other tissues of *Ercc1* ^*Δ/−*^ mice and also identified the increased expression of pathways and genes involved in oxidative stress response, immune response, and downregulation of the IGF‐1 pathway (Karakasilioti et al. 2013; Chatzidoukaki et al. 2021; Schermer et al. 2013; Niedernhofer et al. 2006; Schumacher et al. 2008). This suggests that Ercc1 deficiency has similar effects in the aorta as in tissues with completely different structures and functions such as the liver and kidney, underlining the systemic nature of the aging phenotype in this model. A novel finding specific to the aorta is the phenotypic switch of VSMCs to a macrophage‐like phenotype in the aortic wall of *Ercc1* ^*Δ/−*^ mice. As previously mentioned, macrophage‐like VSMCs acquire phagocytic properties and engage in interactions with (non)resident immune cells, which can trigger a chronic inflammatory state in the aortic wall (Sorokin et al. 2020). Macrophage‐like VSMCs promote inflammation by expressing cytokines such as IL‐1β, IL‐8, IL‐6, and CCL2 and adhesion molecules including VCAM‐1 and ICAM‐1 (Orr et al. 2010). In atherosclerosis, oxLDL and cholesterol are the main metabolic factors that contribute to the differentiation of VSMCs into the macrophage‐like phenotype (Rong et al. 2003). *Ercc1* ^*Δ/−*^ mice do exhibit changes in metabolism but do not develop atherosclerosis. However, our current findings, as well as data from previous studies, suggest that a deficiency in DNA repair could also contribute to this phenotypic switch (Sakai et al. 2023; Skarpengland et al. 2016). In a study with Ku80‐deficient atherosclerotic *ApoE* ^*−/−*^ mice, DNA double‐strand breaks were shown to play a causative role in the induction of a pro‐inflammatory response that precedes the onset of atherosclerosis (Sakai et al. 2023). Furthermore, deficiency of the DNA glycosylase NEIL3, which functions in the repair of oxidized DNA bases, aggravated atherosclerotic plaque development in *ApoE* ^*−/−*^ mice (Skarpengland et al. 2016). More specifically, mice with a smooth muscle cell‐specific knockout of *Ercc1* show elevated plasma levels of the pro‐inflammatory marker IL‐6 (Ataei Ataabadi et al. 2021).

The cGAS‐STING pathway plays an important role in the induction of inflammation in response to DNA damage. The presence of cytosolic DNA as a result of DNA damage is recognized by cGAS as a danger‐associated molecular pattern (DAMP), which subsequently activates STING on the endoplasmic reticulum (ER). Next, STING activates transcription factors that induce downstream expression of type I IFNs and other cytokines such as IL‐6, eliciting an inflammatory response. Activation of the cGAS‐STING pathway has been associated with a number of CVDs, including atherosclerosis, aortic aneurysms, myocardial infarction, and hypertension (An et al. 2024). Additionally, cGAS‐STING activation was linked to phenotypic switching of VSMCs to an inflammatory phenotype in patients with aortic aneurysm and dissection (Abhijit et al. 2023). Independent studies on ERCC1‐deficient non‐small cell lung cancer cells, pancreatic cells, and tissue‐resident microglia have demonstrated that ERCC1 loss leads to an accumulation of cytoplasmic DNA fragments (Chatzidoukaki et al. 2021; Chabanon et al. 2019; Arvanitaki et al. 2024). Our findings provide evidence that this mechanism also occurs in the *Ercc1* ^*Δ/−*^ aorta, as we observe increased expression and predicted activation of cGAS‐STING pathway factors, including STING1, IRF7, and IRF9, along with the presence of cytoplasmic ssDNA, which may contribute to driving this activation. The formation of R‐loops caused by the accumulation of irreparable DNA lesions can trigger the release of cytoplasmic ssDNAs, resulting in cGAS‐STING activation. Previous research reveals the accumulation of cytoplasmic ssDNA caused by R‐loop formation in *Ercc1* ^*−/−*^ pancreas and cells, stimulating a pro‐inflammatory response (Chatzidoukaki et al. 2021). XPF, which forms a heterodimer with ERCC1 to form the active nuclease, is recruited at R‐loop sites and is suggested to be involved in the processing of R‐loops. Thus, ineffective or alternative R‐loop repair caused by Ercc1 deficiency could trigger the release of DNA into the cytoplasm, subsequently resulting in cGAS‐STING activation and the induction of an inflammatory response in the *Ercc1* ^*Δ/−*^ aorta. Moreover, genome‐wide, gene‐length‐dependent transcription stress resulting from the accumulation of transcription‐blocking DNA damage could play a key role here. Previous research shows that there is a significant bias toward the expression of short genes compared to long genes in the liver of *Ercc1* ^*Δ/−*^ mice at 11 weeks of age, supporting genome‐wide accumulation of transcription‐stalling lesions (Vermeij et al. 2016). Besides these findings in DNA‐repair‐deficient mice, gene‐length‐dependent transcription stress is found upon normal aging in many organs and tissues in humans and numerous other species (Soheili‐Nezhad et al. 2024; Gyenis et al. 2023). This universal aging phenomenon is associated with increased R‐loop formation and transcription stress could thereby also promote cGAS‐STING pathway activation (Tresini et al. 2015; Crossley et al. 2019; Zhang et al. 2024).

Additionally, alterations in blood pressure, which affect local hemodynamics and the mechanical load on the aortic wall, and remodeling of the ECM can induce pro‐inflammatory gene expression in VSMCs (Orr et al. 2009; Lehoux et al. 2006). Previous research shows that *Ercc1* ^*Δ/−*^ mice have elevated blood pressure and display increased vascular stiffness (Durik et al. 2012). Moreover, increased ECM remodeling is observed in the *Ercc1* ^*Δ/−*^ aortic wall evidenced by increased MMP activity, elastin fragmentation, and proteoglycan deposition (van der Linden et al. 2024). Thus, these factors could also contribute to increased pro‐inflammatory gene expression and drive the switching of VSMCs to the macrophage‐like phenotype in the *Ercc1* ^*Δ/−*^ aorta. Furthermore, systemic inflammatory factors may further promote this phenotypic switch. Ercc1 deficiency triggers a pro‐inflammatory immune response in adipose tissue, the kidney, and the pancreas (Karakasilioti et al. 2013; Chatzidoukaki et al. 2021; Schermer et al. 2013) and deficiency of Ercc1 exclusively in the immune system causes the premature onset of immunosenescence (Yousefzadeh et al. 2021). This raises the question of whether the phenotypic switching of VSMCs in *Ercc1* ^*Δ/−*^ mice is driven primarily by DNA damage within VSMCs or by systemic factors. Research on endothelial cell‐specific (EC‐KO) and smooth muscle cell‐specific (SMC‐KO) ERCC1 knockout mice has confirmed cell type‐specific contributions to the vascular aging process, with both models displaying increased aortic stiffness and impaired vasodilation (Ataei Ataabadi et al. 2021; Bautista‐Niño et al. 2020). Notably, SMC‐KO mice also exhibited elevated plasma cytokine levels. These findings indicate that ERCC1 deficiency in either ECs or SMCs is sufficient to induce vascular dysfunction, which is indicative of VSMC phenotypic switching. Further research is needed to confirm a direct link between ERCC1 deficiency and phenotypic switching in VSMCs, which could be explored by further investigation of the SMC‐KO model or studying the effects of DNA damage on the phenotype of ERCC1‐deficient VSMCs in vitro.

DR has been shown to effectively delay the process of aging and increase life expectancy in animals as well as in humans. More specifically, DR attenuates multiple characteristics of cardiovascular aging, including oxidative stress, vascular inflammation, endothelial dysfunction, and vascular stiffness (Weiss and Fontana 2011). The accumulation of DNA damage is suggested to be an important driver in the process of cardiovascular aging, which is evidenced by the fact that DNA‐repair‐deficient *Ercc1* ^*Δ/−*^ mice portray multiple characteristics of cardiovascular aging (van der Linden et al. 2024). DR has previously shown promising results in *Ercc1* ^*Δ/−*^ mice, delaying characteristics of aging including disturbed motor function, neurodegeneration, and osteoporosis, and drastically extending their lifespan (Durik et al. 2012). We now show that DR also functionally and structurally improves cardiovascular health in *Ercc1* ^*Δ/−*^ mice. Besides improving cardiac function in vivo, by increasing left ventricle stroke volume, our results demonstrate that DR alleviates aspects of vascular aging in the *Ercc1* ^*Δ/−*^ aorta, including ECM remodeling, oxidative stress, and inflammation. Notably, DR also decreases the expression of markers for the macrophage‐like VSMC phenotype, which possibly contributes to an inflammatory response in the *Ercc1* ^*Δ/−*^ aortic wall. Additionally, we show that DR decreased STING1 expression in the *Ercc1* ^*Δ/−*^ aorta, which might be causative in the induction of the macrophage‐like VSMC phenotype. DR is suggested to preserve genomic function by alleviating endogenously generated DNA damage and thereby reducing genome‐wide transcription stress, which would subsequently result in less activation of the cGAS‐STING pathway and thereby attenuate the inflammatory response in the *Ercc1* ^*Δ/−*^ aorta (Vermeij et al. 2016; Heydari et al. 2007). At this stage, it remains unclear whether DR directly modulates the identified vascular aging processes or if the observed changes result from broader systemic effects. To distinguish between cell‐intrinsic and systemic effects, vessel‐on‐a‐chip models or in vitro studies using VSMCs could be valuable tools.

Given the challenges of implementing DR as a therapeutic strategy for vascular aging, we explored alternative compounds that could mimic its beneficial effect on the aorta. Our RNAseq analysis identified rapamycin and resveratrol, both known DR mimetics with therapeutic potential for CVD. Furthermore, losartan and PPAR agonists pirixinic acid and troglitazone were identified as possible therapeutic compounds. Although these compounds show therapeutic potential, they are yet to be tested in the context of vascular aging induced by DNA repair deficiency. Notably, UR analysis also identified TD139, a MAC2 inhibitor shown to suppress NLRP3 inflammasome activation in macrophages and exert anti‐atherogenic effects in *ApoE* ^*−/−*^ mice (Li et al. 2021). This suggests that TD139 may counteract the DNA damage‐induced immune response that accelerates vascular aging in the *Ercc1* ^*Δ/−*^ aorta. In conclusion, besides the beneficial effect on longevity and general health span, DR also positively affects cardiovascular health specifically. Future research is needed to evaluate the therapeutic potential of these compounds in vascular aging.

## Author Contributions

S.J.M.S., J.H.J.H., R.K., J.E., and I.P. conceptualized and designed the project. S.J.M.S. and N.V. isolated RNA from aorta tissue. J.M.H.‐G. and S.J.M.S. analyzed RNA sequencing data. S.J.M.S., J.L., and D.S. performed (immuno)histological analysis on the aorta. S.J.M.S. and M.B. performed in vitro experiments with VSMCs. J.L. and J.H.M.O. performed immunoblot analysis. N.V. and I.N.‐B. performed ultrasound measurements and N.V. and S.J.M.S. analyzed ultrasound data. R.M.C.B. and S.B. managed mouse work. Y.R. assisted with molecular imaging experiments. S.J.M.S., J.L., J.M.H.‐G., J.H.J.H., R.K., J.E., and I.P. wrote and edited the manuscript.

## Conflicts of Interest

The authors declare no conflicts of interest.

## Supporting information


Data S1.

## Data Availability

The data discussed in this publication have been deposited in NCBI's Gene Expression Omnibus and are accessible through GEO Series accession number GSE294678 (Edgar 2002).
