# Supplementary material for: Dietary Restriction Mitigates Vascular Aging, Modulates the cGAS‐STING Pathway and Reverses Macrophage‐Like VSMC Phenotypes in Progeroid DNA‐Repair‐Deficient Ercc1Δ /− Mice
Source: Aging Cell. 2025 Apr 25;24(7):e70062. doi: 10.1111/acel.70062 (PMC12266768; doi:10.1111/acel.70062)
Supplement: Supplementary file 1 — Data S1. [file ACEL-24-e70062-s001.pdf]

***Table 1. Number and sex of mice used in each experimental group for mRNA sequencing.***

| | 6 wks | 22 wks | |
|---|---|---|---|
| | AL | AL | DR |
| *WT* | N = 5 (3M, 2F) | N = 4 (3M, 1F) | N = 5 (3M, 2F) |
| *Ercc1* | N = 5 (3M, 2F) | N = 4 (2M, 2F) | N = 5 (3M, 2F) |

*Table 2. Antibody conditions used in the indicated experiments.*

| Method | Primary antibody | Product number | Company | Dilution | Secondary antibody | Company | Dilution |
|---|---|---|---|---|---|---|---|
| **Immunohistochemical staining** | Anti-MAC2 | CL8942AP | Cedarlane | 1:1500 | Biotinylated α-rat IgG | DAKO | 1:200 |
| | Anti-VCAM1 | Ab134047 | Abcam | 1:500 | Biotinylated α-rabbit IgG | DAKO | 1:200 |
| **Immunofluorescent staining** | Anti-STING | 19851-1-AP | Proteintech | 1:500 | α-rabbit alexa 594 | Molecular Probes | 1:1000 |
| | Anti-cGAS | 26416-1-AP | Proteintech | 1:200 | α-rabbit alexa 488 | Molecular Probes | 1:1000 |
| | Anti-ssDNA | MAB3299 | Sigma-Aldrich | 1:100 | α-mouse alexa 488 | Molecular Probes | 1:1000 |
| | Anti-MYH11 | Ab82541 | Abcam | 1:500 | α-rabbit alexa 594 | Molecular Probes | 1:1000 |
| | Anti-αSMA | Ab7818 | Abcam | 1:750 | α-mouse alexa 488 | Molecular Probes | 1:1000 |
| **Western blot** | Anti-VCAM1 | Ab134047 | Abcam | 1:1000 | HRP-conjugated α-rabbit IgG | Biolegend | 1:1000 |
| | Anti-MMP3 | Ab52915 | Abcam | 1:500 | HRP-conjugated α-rabbit IgG | Biolegend | 1:1000 |
| | Anti-MAC2 | CL8942AP | Cedarlane | 1:1000 | HRP-conjugated α-rat IgG | DakoCytomation | 1:1000 |
| | Anti-TIMP1 | MA5-13688 | Thermo Scientific | 1:1000 | HRP-conjugated α-mouse IgG | Jackson Immunoresearch | 1:1000 |
| | Anti-STING | 13647S | Cell Signaling | 1:500 | HRP-conjugated α-rabbit IgG | Biolegend | 1:1000 |

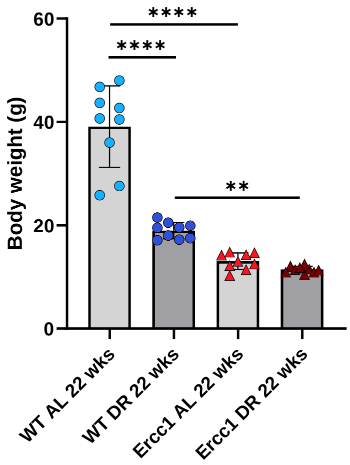


***Figure 1. Body weight of Ercc1$^{\Delta/-}$ and WT mice on AL or DR at 22 weeks of age.*** The mean ± SD is plotted (n = 9 per group, two-way ANOVA, **p<0.01, ****p<0.0001).

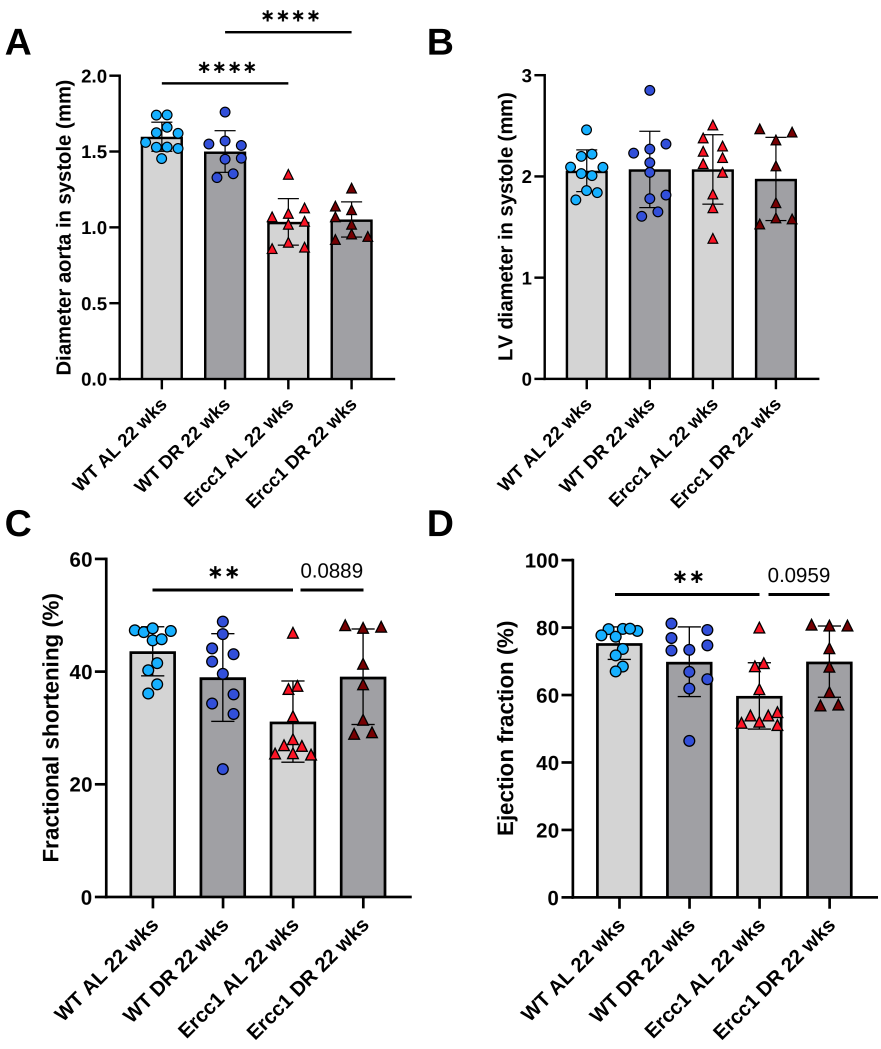


***Figure 2.*** **(A)** Aorta diameter in systole and **(B)** LV diameter in diastole of *Ercc1*$^{\Delta/-}$ and WT mice on AL or DR at 22 weeks of age. The average value of 3 individual measurements is plotted per mouse (mean ± SD, n = 8-10 per group, two-way ANOVA, ****p<0.0001). **(C)** Fractional shortening (the percentage change in the LV diameter between diastole and systole) and **(D)** ejection fraction (the percentage of blood that is pumped out of the LV with each cardiac cycle) derived from M-mode images of the left ventricle of *Ercc1*$^{\Delta/-}$ and WT mice on AL or DR at 22 weeks of age. The average value of 3 individual measurements is plotted per mouse (mean ± SD, n = 8-10 per group, two-way ANOVA, **p<0.01).

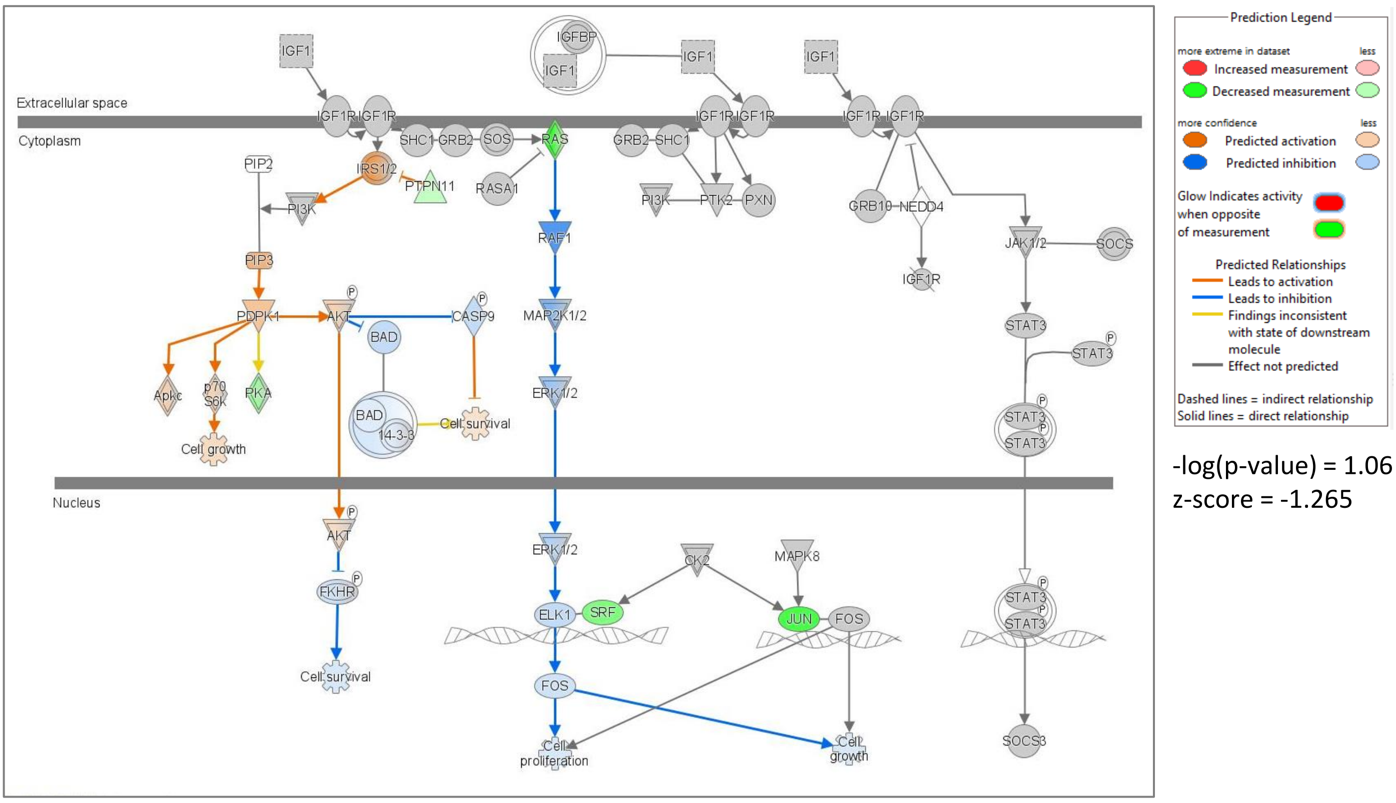


***Figure 3. Expression of the IGF-1 signaling pathway in the aorta of 22-week-old Ercc1$^{\Delta/-}$ mice compared to WT mice.*** Ingenuity Pathway Analysis (IPA) reveals downregulation of *Jun, Pka, Ptpn11, Ras* and *Srf* and predicted downstream inhibition of the IGF-1 signaling pathway.

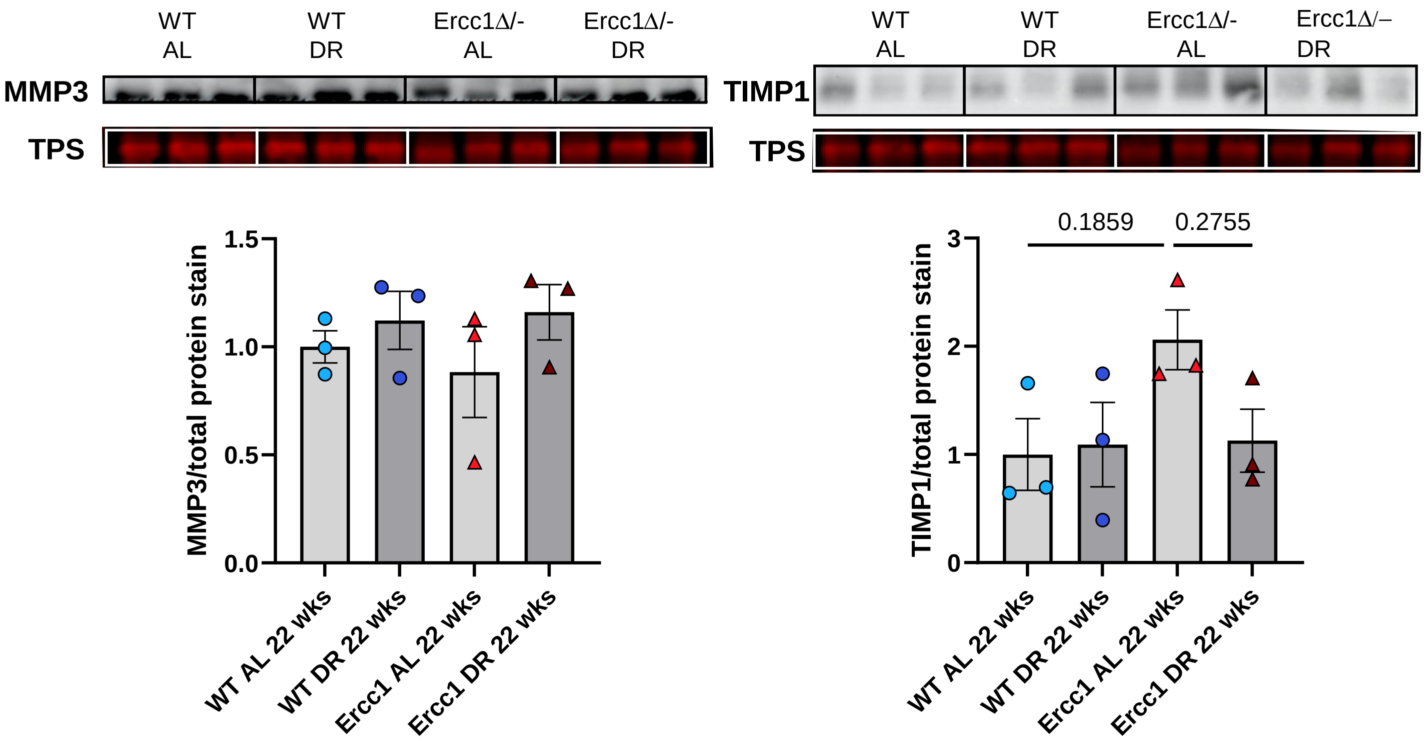


***Figure 4.*** Western blot showing MMP3 and TIMP1 protein expression in *Ercc1*$^{\Delta/-}$ and WT mouse aorta. MMP3 and TIMP1 protein levels were corrected for total protein (Total Protein Stain). The average of 2 separate Western blots was plotted per mouse (mean ± SD, n = 3 per group, two-way ANOVA).

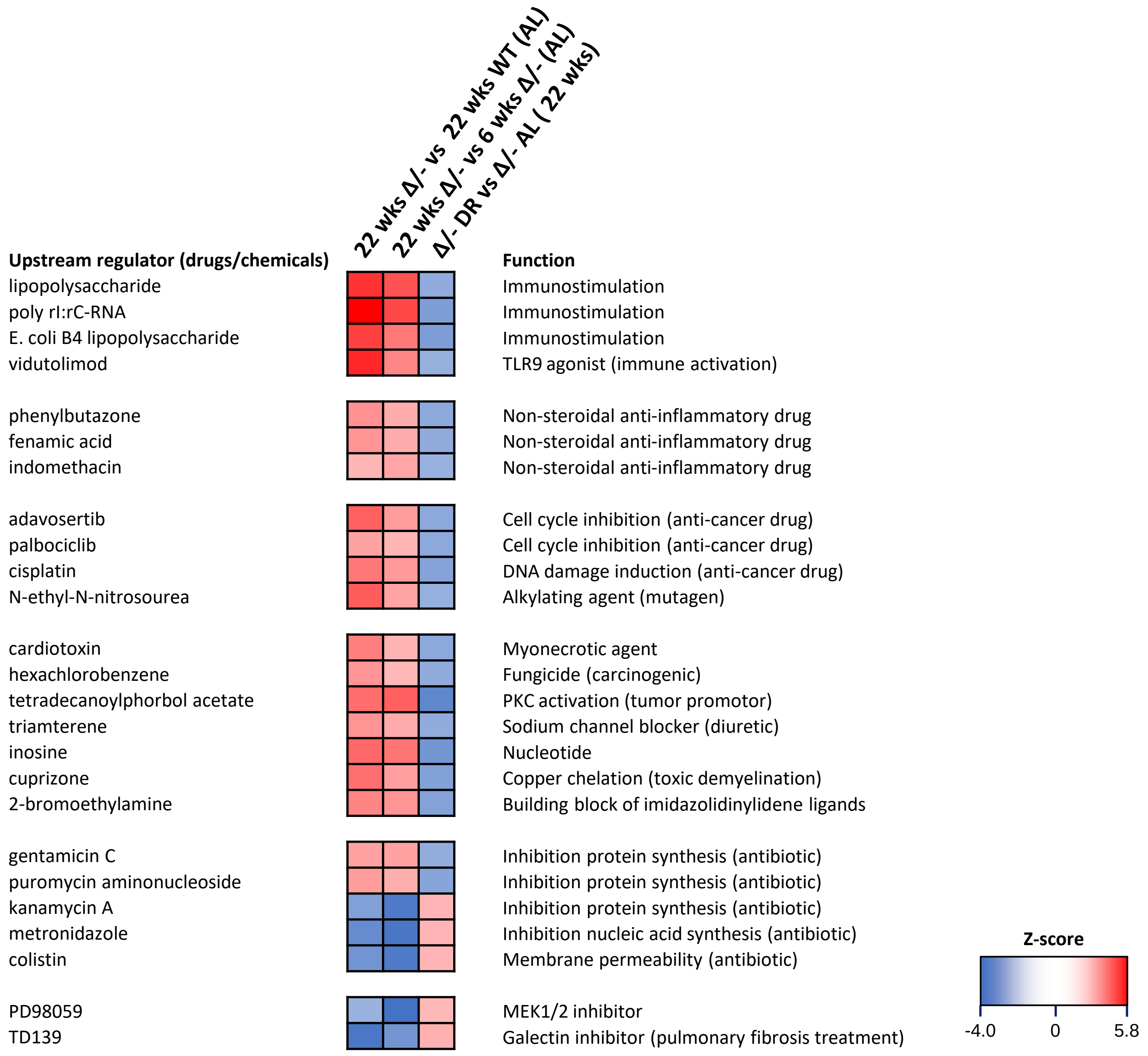


***Figure 5. Upstream Regulator analysis of drugs and chemicals that switch direction upon dietary restriction.*** URs were selected that were either activated or inhibited in both of the following comparisons; 22-week-old Ercc1$^{\Delta/-}$ vs WT aorta (AL diet) and 22-week-old Ercc1$^{\Delta/-}$ vs 6-week-old Ercc1$^{\Delta/-}$ aorta. Subsequently, URs were selected that switched direction (from activated to inhibited or from inhibited to activated) when compared to the Ercc1$^{\Delta/-}$ on DR vs Ercc1$^{\Delta/-}$ on AL comparison. Z-score indicates whether an UR is predicted to be activated or inhibited based on the expression fold changes of its downstream genes.

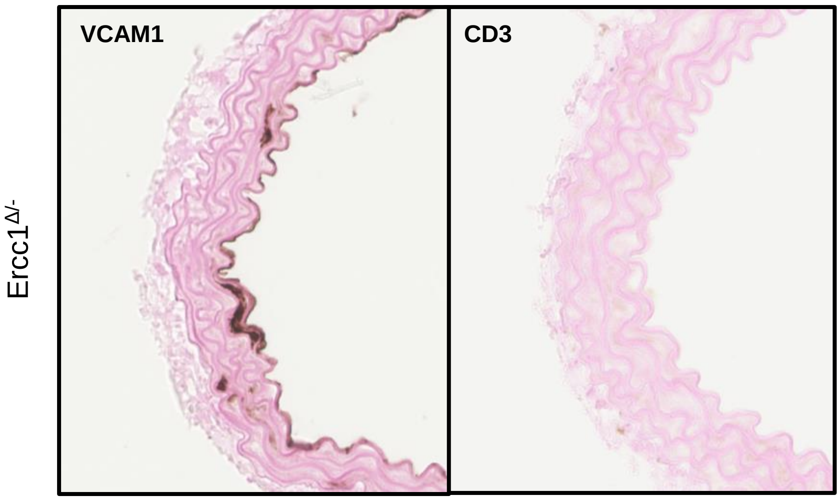


***Figure 6. Representative images of immunohistochemical staining for VCAM1 (left) and CD3 (right) on the same aortic ring of a Ercc1$^{\Delta/-}$ mouse.***

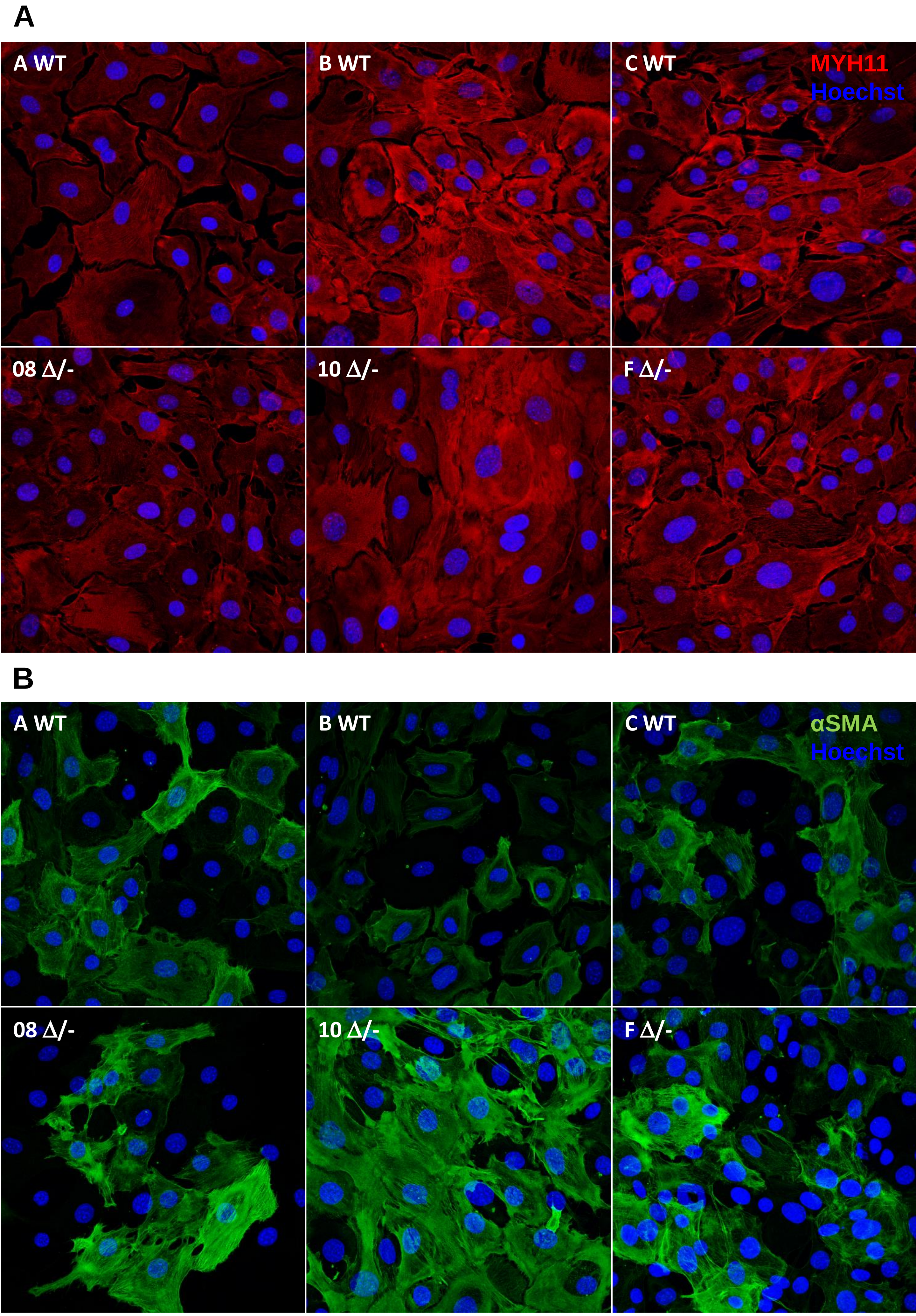


***Figure 7. (A)*** *Representative images of immunofluorescent staining for MYH11 on VSMCs isolated from the aorta of Ercc1$^{+/+}$ and Ercc1$^{\Delta/-}$ mice (n=3 per genotype).* ***(B)*** *Representative images of immunofluorescent staining for αSMA on VSMCs isolated from the aorta of Ercc1$^{+/+}$ and Ercc1$^{\Delta/-}$ mice (n=3 per genotype).*

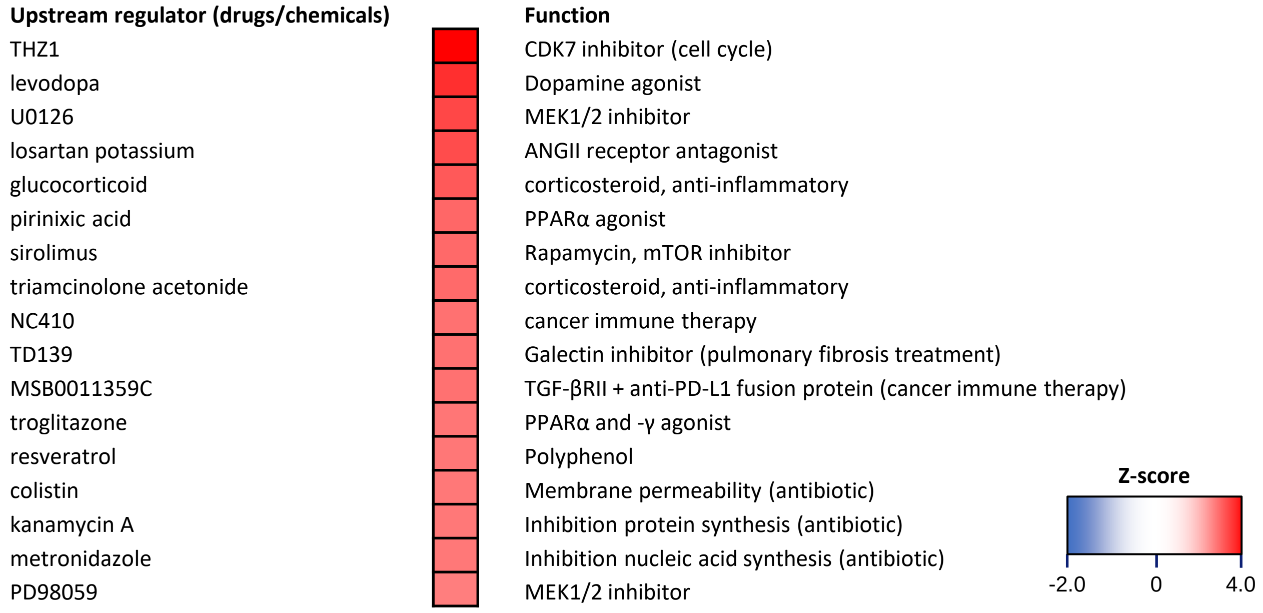


***Figure 8. Upstream Regulator analysis of drugs and chemicals predicted to be activated in the aorta of Ercc1$^{\Delta/-}$ mice on dietary restriction compared to Ercc1$^{\Delta/-}$ mice on ad libitum diet.***
